# Development of [^18^F]AldoView as the First Highly Selective Aldosterone Synthase PET Tracer for Imaging of Primary Hyperaldosteronism

**DOI:** 10.1021/acs.jmedchem.1c00539

**Published:** 2021-06-17

**Authors:** Kerstin Sander, Thibault Gendron, Klaudia A. Cybulska, Fatih Sirindil, Junhua Zhou, Tammy L. Kalber, Mark F. Lythgoe, Tom R. Kurzawinski, Morris J. Brown, Bryan Williams, Erik Årstad

**Affiliations:** † Centre for Radiopharmaceutical Chemistry, University College London, 5 Gower Place, London WC1E 6BS, U.K.; ‡ William Harvey Research Institute, Barts & The London School of Medicine & Dentistry, Queen Mary University of London, Charterhouse Square, London EC1M 6BQ, U.K.; § Centre for Advanced Biomedical Imaging, University College London, 72 Huntley Street, London WC1E 6DD, U.K.; ∥ NIHR University College London Hospitals Biomedical Research Centre, 149 Tottenham Court Road, London W1T 7DN, U.K.; ⊥ Institute of Cardiovascular Sciences, University College London, Gower Street, London WC1E 6BT, U.K.

## Abstract

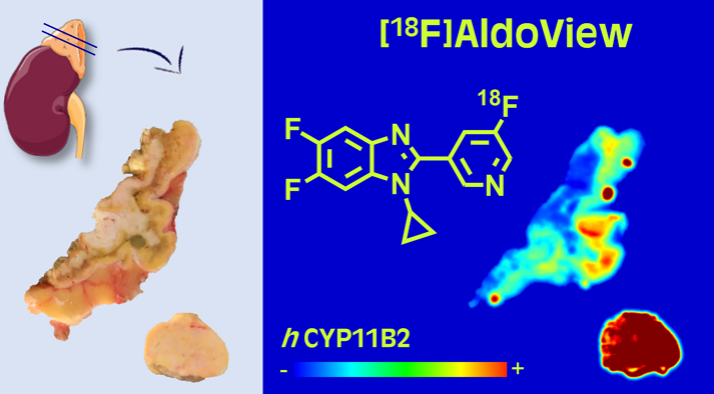

The purpose of this study was to synthesize a fluorine-18 labeled, highly selective aldosterone synthase (*h*CYP11B2) inhibitor, [^18^F]AldoView, and to assess its potential for the detection of aldosterone-producing adenomas (APAs) with positron emission tomography in patients with primary hyperaldosteronism (PHA). Using dibenzothiophene sulfonium salt chemistry, [^18^F]AldoView was obtained in high radiochemical yield in one step from [^18^F]fluoride. In mice, the tracer showed a favorable pharmacokinetic profile, including rapid distribution and clearance. Imaging in the adrenal tissue from patients with PHA revealed diffuse binding patterns in the adrenal cortex, avid binding in some adenomas, and “hot spots” consistent with aldosterone-producing cell clusters. The binding pattern was in good visual agreement with the antibody staining of *h*CYP11B2 and distinguished areas with normal and excessive *h*CYP11B2 expression. Taken together, [^18^F]AldoView is a promising tracer for the detection of APAs in patients with PHA.

## Introduction

Hypertension is a leading cause of premature morbidity and death globally. Although lifestyle changes and medication can be effective, elevated blood pressure due to secondary causes is more difficult to control and has greater morbidity. This is particularly true for primary hyperaldosteronism (PHA), which is characterized by excessive, autonomous aldosterone production by the adrenals.^1^ PHA occurs in 5–10% of patients with hypertension and in 15–25% of those with treatment-resistant hypertension.^2^ Although PHA is a potentially curable cause of secondary hypertension, it is estimated that >99% of those with PHA remain undiagnosed and on lifelong medication at a high cost to individuals and healthcare budgets.^3^ In most cases, PHA is due to either bilateral hyperplasia of the adrenal cortex or an aldosterone-producing adenoma (APA). For patients with a demonstrable unilateral cause, surgical removal of the abnormal gland (adrenalectomy) often results in reduced blood pressure, lower dependence on antihypertensive drugs, and sometimes in the complete cure of hypertension (30–60% of cases).^4^ The challenge is how to identify such patients.

Many patients who are >50 years old have adenomas as incidental findings; yet, these are not necessarily a source of excess aldosterone production.^5^ Conversely, some APAs are smaller than the limit of detection for computed tomography (CT) or magnetic resonance imaging (MRI) and may be contralateral to an adenoma. This recognition of frequent microadenomas (diameter <1 cm) requires techniques to detect minute functional lesions, in order to identify a surgical opportunity to resect the affected adrenal on one side, and not miss a cause of persistent PHA postadrenalectomy on the other.^6−8^ To avoid removing the wrong adrenal, the current diagnostic pathway to lateralize patients with PHA involves the following: biochemical tests that suggest autonomous, excess aldosterone production, followed by the identification of an adrenal nodule consistent with an adenoma by CT or MRI, followed by adrenal vein sampling (AVS) to assess whether the adrenal nodule is a source of excess aldosterone production. However, AVS is technically challenging, invasive, not widely available, and is often not feasible as it requires patients with severe hypertension to adjust or stop medication for several weeks.^9^ This complex diagnostic route is the major reason why so few patients ultimately undergo adrenalectomy, a potentially curative procedure for their hypertension.

Inappropriately high aldosterone production in patients with PHA is due to the increased aldosterone synthase (CYP11B2) activity. CYP11B2 is the only known source of aldosterone production, and a large body of evidence shows overexpression of this enzyme in adrenal tissue from patients with PHA (often >10–100 fold). For these reasons, evidence of abnormal CYP11B2 expression in adrenal lesions, or the surrounding adrenal cortex, is considered both necessary and sufficient for the histopathological diagnosis of PHA.^10^

Imaging of CYP11B2 with positron emission tomography (PET) is an appealing alternative to the aforementioned diagnostic pathway as it could enable the identification and lateralization of adrenal glands with areas of excessive aldosterone production and reveal the functional status of adrenal nodules detected with CT or MRI. However, selective in vivo imaging of CYP11B2 has not yet been achieved due to the close homology between the enzymes involved in aldosterone (CYP11B2) and cortisol (CYP11B1) synthesis.^11−18^ This is a major limitation as CYP11B1 is highly expressed in healthy adrenals and some adenomas, without any apparent correlation with the rate of aldosterone excretion.

Recently, Merck & Co. reported a class of highly selective aldosterone synthase inhibitors, derived from benzimidazole.^19^ Using a dibenzothiophene sulfonium salt—a new fluorine-18 chemistry platform developed by our group^20^—we labeled the benchmark benzimidazole ligand to give the first highly selective CYP11B2 PET tracer (aka [^18^F]AldoView; IC_50_ 4.7 nM vs 435 nM for CYP11B1). Herein, we describe the synthesis of [^18^F]AldoView and its characterization in preclinical studies.

## Results

### Chemistry

**Scheme 1:**
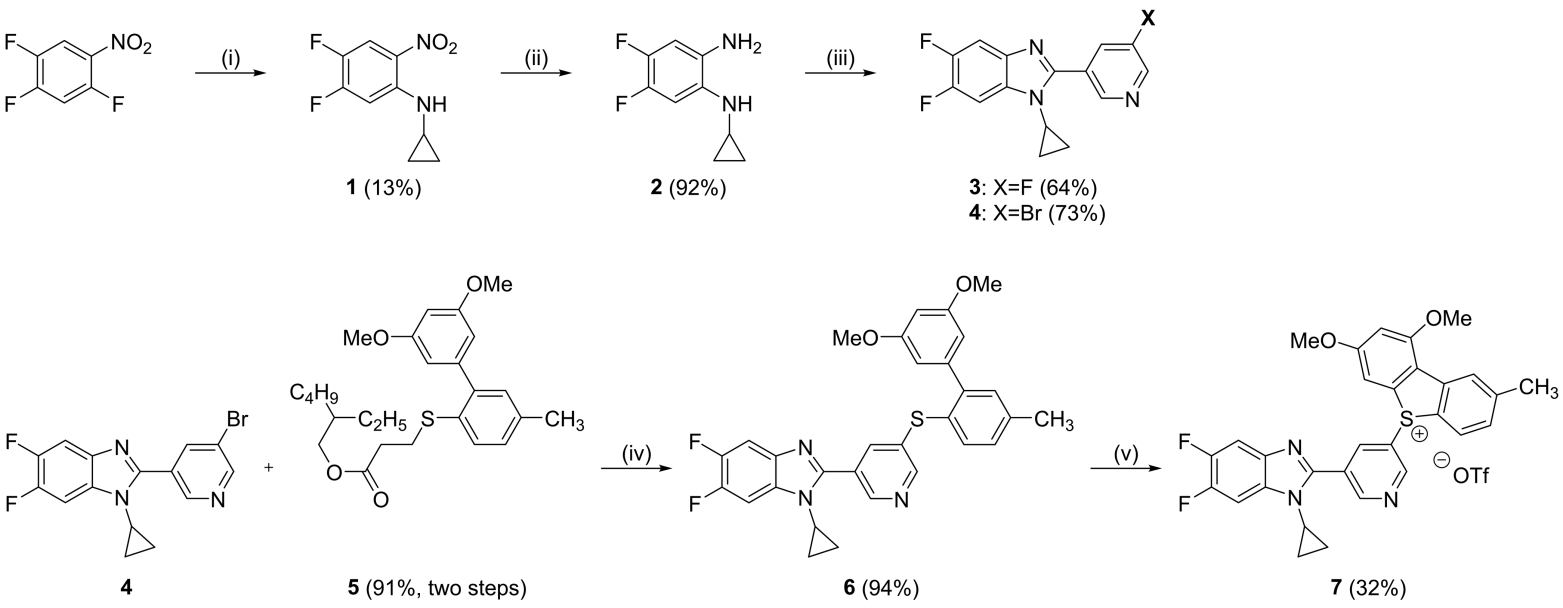
Synthesis of the [^18^F]AldoView Nonradioactive Reference Compound **3** and the Labeling Precursor **7** Reagents and conditions: (i) cyclopropylamine, trimethylamine, tetrahydrofuran, 60 °C, 2 h; (ii) Pd/C, H_2_, methanol, rt, 20 h; (iii) 5-fluoronicotinaldehyde (for **3**)/5-bromonicotinaldehyde (for **4**), oxone, dimethylformamide/water, rt, 1 h; (iv) Pd_2_(dba)_3_, DPEphos, toluene, rt, 10 min; **5**, C_4_H_9_KO, toluene, 125 °C, 2 h; and (v) NaOTf, Ca(OCl)_2_, acetate buffer (pH = 4), acetone, 0 °C—rt, 1 h.

The nonradioactive benchmark compound **3** was synthesized following previously reported procedures (Scheme 1).^19,21^ The [^18^F]AldoView precursor for labeling with fluorine-18 was prepared in three steps starting from the diamine **2**. Condensation of **2** with 5-bromonicotinaldehyde in the presence of oxone gave the bromo-pyridine **4** in 73% yield. Subsequent Suzuki cross-coupling of **4** with the protected biaryl **5** (prepared as described previously)^20^ provided the thioether **6** in 94% yield. Ring closure of **6** initiated by calcium hypochlorite led to the formation of the dibenzothiophene sulfonium salt **7**. The purity of the labeling precursor **7** was >97% as determined by high-performance liquid chromatography (HPLC) (Figure S1).

### Radiochemistry

**Scheme 2:**
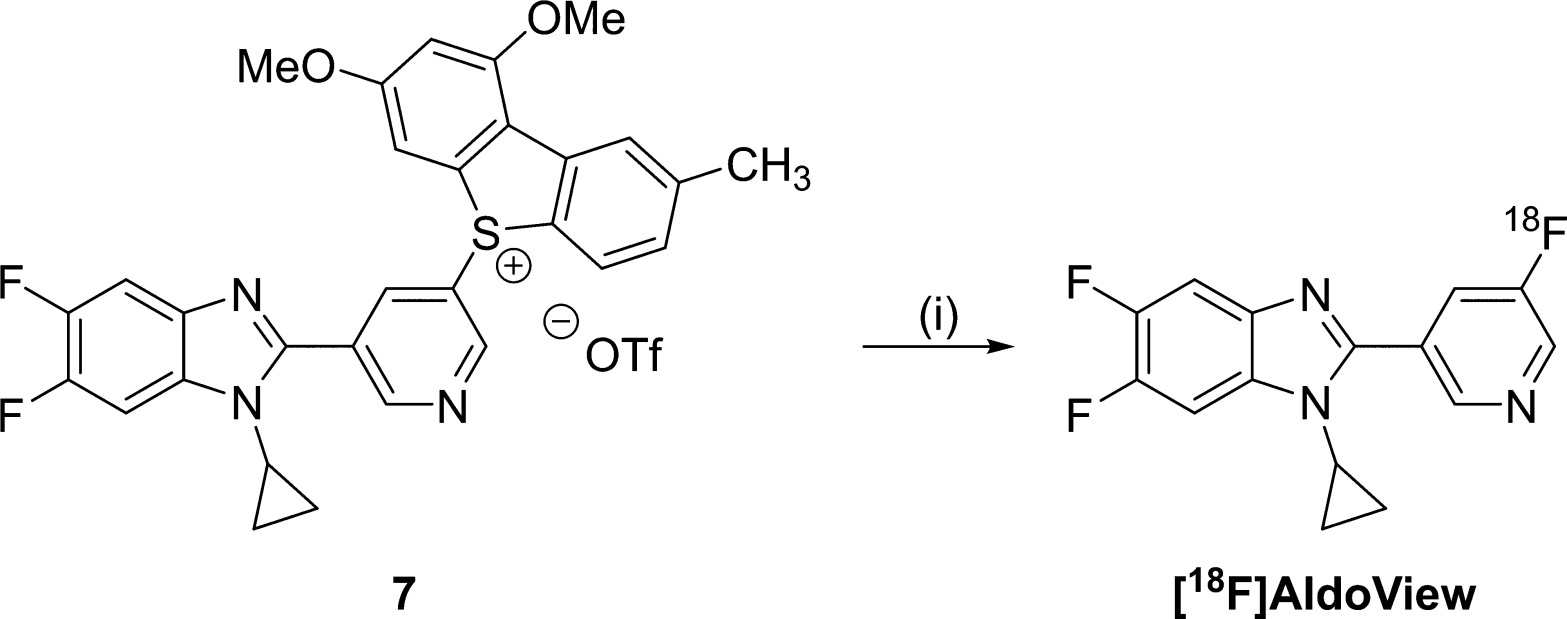
Radiolabeling of [^18^F]AldoView Reagents and conditions: (i) [^18^F]fluoride in [^18^O]water, Kryptofix 222, KHCO_3_, dimethyl sulfoxide, 110 °C, 15 min.

The reaction of the dibenzothiophene sulfonium salt **7** with [^18^F]fluoride was carried out using a low precursor load of 2 mg per reaction. The reaction conditions were designed to suit automated radiosynthesis following good manufacturing practice (Scheme 2).^22^ [^18^F]AldoView was obtained in 42 ± 8% (decay-corrected; *n* = 12) radiochemical yield, with a radiochemical purity of >99%, and with a molar activity of 31 ± 3 GBq/μmol (*n* = 4) when starting from 2 GBq of [^18^F]fluoride (Figure S2).

### PET/CT Imaging

**Figure 1:**
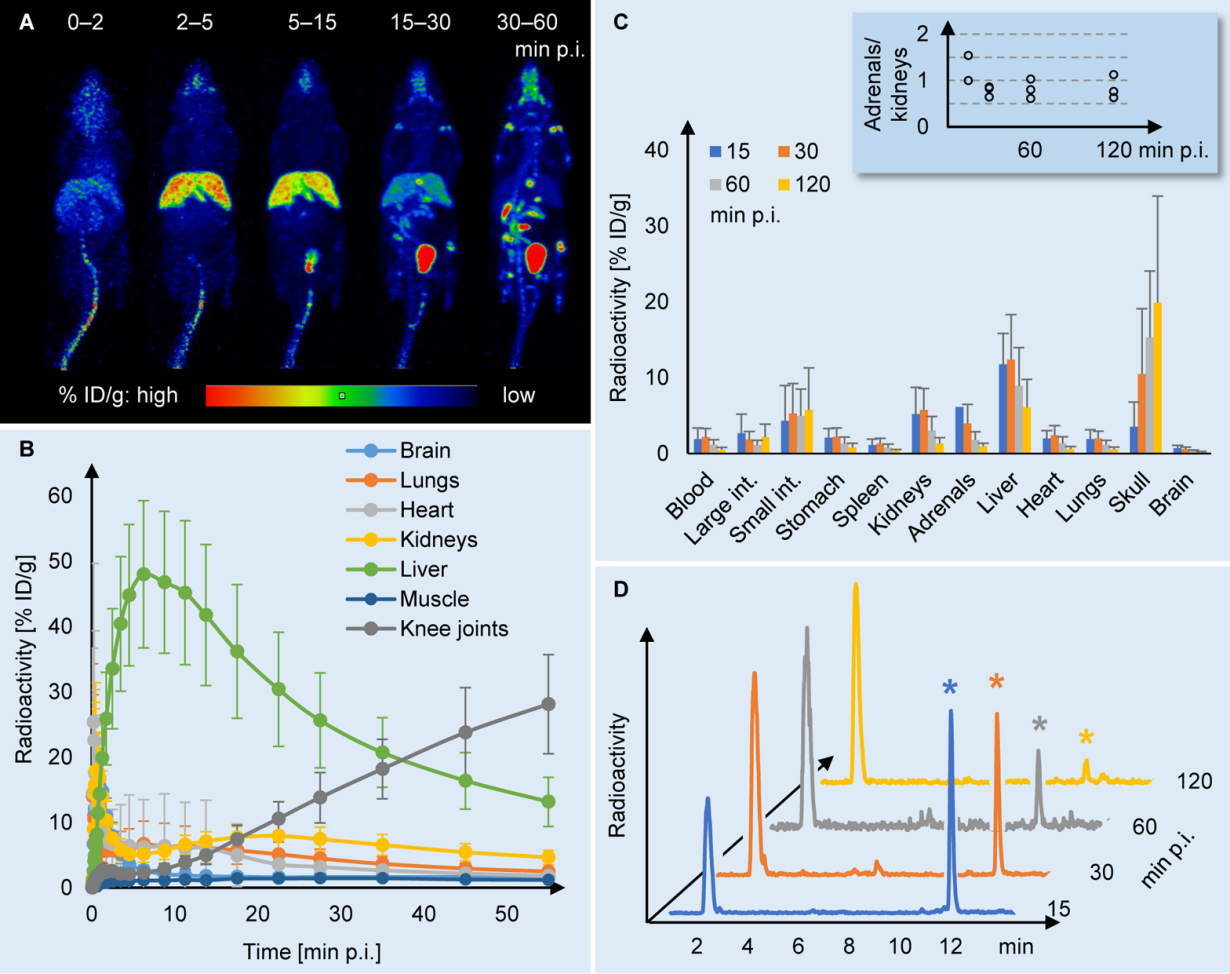
In vivo and ex vivo assessment of the CYP11B2-selective PET tracer [^18^F]AldoView. (A) Summed PET/CT images representing five different time frames of a dynamic scan with [^18^F]AldoView in healthy female BALB/c mice (scale bar indicates percent injected dose per gram [% ID/g] tissue). (B) PET time–activity curves for [^18^F]AldoView (% ID/g tissue) in female BALB/c mice (*n* = 6). (C) [^18^F]AldoView tissue distribution profile in female BALB/c mice (*n* = 3–4/group). Inset: adrenal-to-kidney ratio of [^18^F]AldoView binding. (D) Representative radio-HPLC chromatograms depicting the plasma metabolite profile in female BALB/c mice (*n* = 2–3/group).

Whole-body imaging of [^18^F]AldoView in female BALB/c mice (*n* = 6) showed a marked liver uptake at the earlier time points (2–5 and 5–15 min, summation images, Figure 1A). At 15–30 min, the distribution was dominated by the activity in the bladder, and at the later time point (30–60 min), uptake in the bone tissue became evident. Dynamic analysis (Figure 1B) showed that the tracer uptake in major organs such as the brain, lungs, and heart peaked at 1–2 min postinjection and then rapidly decreased. In muscle (region of interest across knee extensors), the level of activity remained low (<1.5% ID/g) throughout the duration of the scan (1 h). The kidney time–activity curve appeared to be biphasic, indicating initial perfusion at 0–5 min postinjection, followed by low and constant radioactivity levels of 5–8% ID/g thereafter. By far the highest level of activity was observed in the liver, which peaked at 6 min postinjection with 48 ± 11% ID/g and subsequently decreased to half the peak value within 30 min. At later time points, the bone surpassed the liver as the tissue with the highest levels of activity. No dynamic data were recorded for the adrenals due to the minuscule size of these organs in mice.

### Biodistribution

[^18^F]AldoView was administered by tail vein injection in healthy female BALB/c mice (*n* = 3–4 per time point), and the distribution of radioactivity was measured in selected organs at 15, 30, 60, and 120 min postinjection (Figure 1C). At the 15 min time point, the highest activity levels were observed in the liver (11.8 ± 4.1% ID/g), followed by the adrenals (6.1% ID/g, *n* = 2), kidneys (5.2 ± 3.5% ID/g), and small intestines (4.3 ± 4.7% ID/g). The activity levels in blood and the other organs investigated (stomach, spleen, heart, lungs, brain, and bone) were comparatively low. In the adrenals, the activity levels gradually decreased over time (4.0 ± 2.5% ID/g at 30 min to 1.8 ± 1.1% ID/g at 60 min). The uptake in the kidneys and liver peaked at 30 min (5.7 ± 2.9 and 12.4 ± 5.9% ID/g, respectively) and then cleared, more rapidly so from the kidneys than from the liver. The ratio between the tracer uptake in the adrenals and kidneys was 1.2 at 15 min postinjection and subsequently equilibrated at 0.6–0.7 (inset in Figure 1C). The activity levels in the intestines remained practically unchanged over the course of the experiment (4–6% ID/g). In bone, the activity levels increased from 15 to 60 min, suggesting that the tracer may undergo metabolic defluorination in vivo.

### Metabolite Analysis

To assess the in vivo stability of [^18^F]AldoView, the metabolic profile in blood was determined using radio-HPLC (Figure 1D). At 15, 30, and 60 min postinjection, the intact parent fractions of the tracer were 59, 29, and 21%, respectively. The tracer was still detectable in blood at 120 min, although at low levels. A single, highly polar metabolite accounted for the remaining activity throughout the course of the experiment.

### Human Tissue Imaging

**Figure 2:**
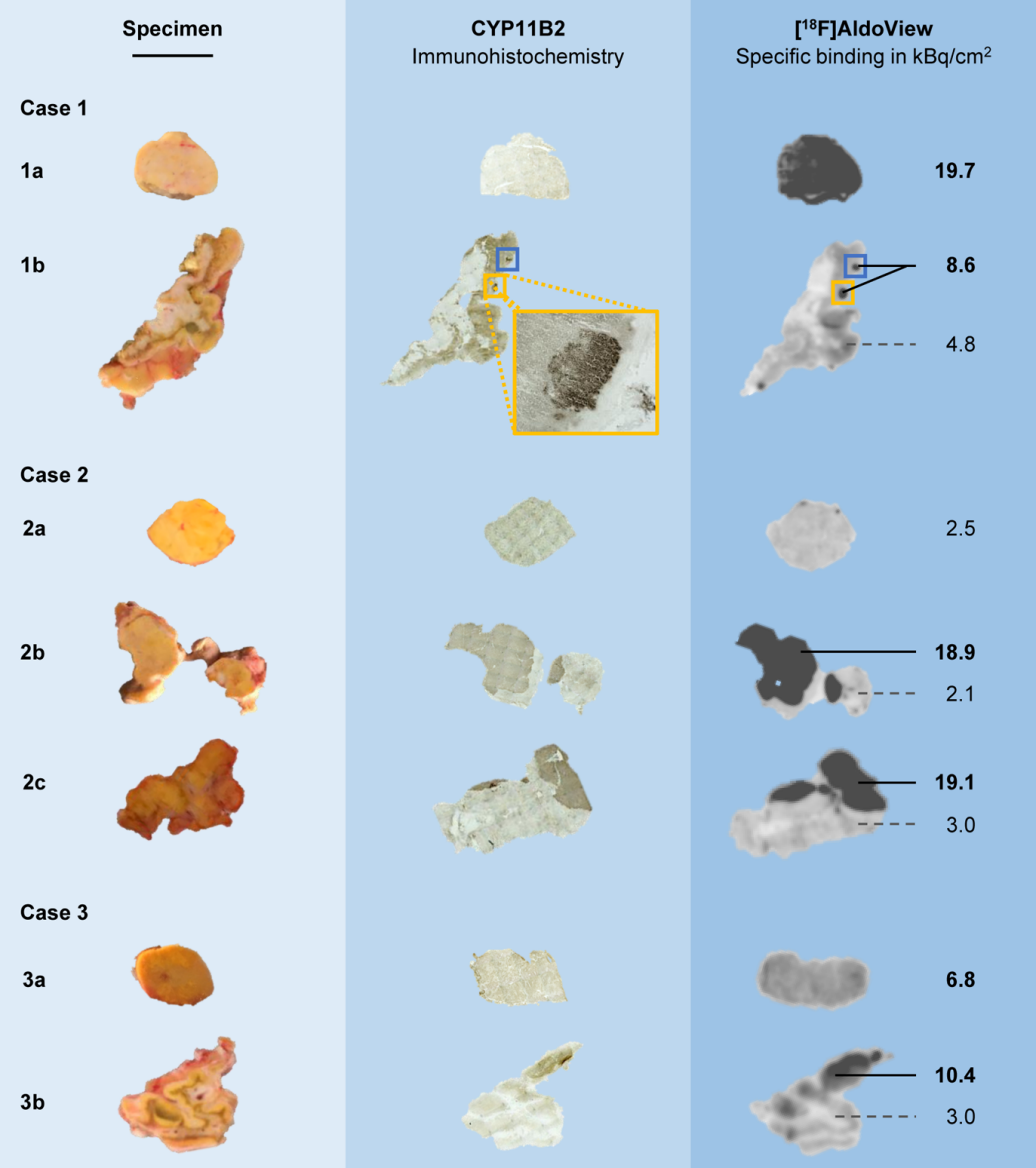
In vitro autoradiography with [^18^F]AldoView in tissue sections from surgically resected adrenal glands in comparison with CYP11B2-specific IHC staining in directly adjacent tissue sections. Case 1 is a female PHA patient aged 41 at the time of adrenalectomy, diagnosed with a 17 × 12 mm adenoma and otherwise normal parenchyma (as determined by CT imaging). Autoradiography revealed a high CYP11B2 expression in the APA (specimen 1a) and in APCCs located in the zona glomerulosa of the adrenal cortex (1b). The tracer binding pattern was in good visual agreement with CYP11B2-specific IHC. Case 2 is a male PHA patient aged 55, diagnosed with a 10 mm adenoma and hyperplasia. [^18^F]AldoView binding comparable to that found in the control tissue was observed in the suspected APA (specimen 2a), suggesting low CYP11B2 expression levels. CYP11B2 IHC was inconclusive due to a lack of contrast. Parts of specimen 2b were identified as APA following CYP11B2 IHC, and subsequent autoradiography revealed high enzyme expression levels. Specimen 2c showed a high CYP11B2 expression adjacent to the adrenal cortex, as determined with autoradiography. The tracer binding pattern was consistent with CYP11B2 IHC. Case 3 is a female PHA patient aged 37 at the time of adrenalectomy, diagnosed with a 9 mm adenoma. [^18^F]AldoView binding to the APA was found to be moderately increased (specimen 3a). An area in specimen 3b, adjacent to the adrenal cortex, revealed high CYP11B2 expression levels by [^18^F]AldoView autoradiography and CYP11B2 IHC. Scale bar: 1 cm. NB1: The different shades of brown in the CYP11B2 IHC staining do not reflect enzyme expression levels. NB2: Autoradiography for cases 4–8 can be found in Figure S3.

The ability of [^18^F]AldoView to depict CYP11B2 expression was assessed by quantitative phosphorimaging in human tissue sections from surgically resected adrenal glands from five patients diagnosed with PHA. Tissue sections from three patients with CYP11B2-negative adrenal lesions were included as controls. Where feasible, several surgical specimens were collected from each patient to include the respective lesions (adenoma/tumor), as well as the adrenal cortex. The expression patterns of CYP11B2 were confirmed independently using immunohistochemical (IHC) staining in adjacent tissue sections. The binding of [^18^F]AldoView (Figures 2, S3) was visually consistent with the IHC staining of CYP11B2 and demarcated areas with dense expression in some of the larger adenomas (e.g., specimens 2b/c in Figure 2), adrenal cortex (e.g., specimen 3b in Figure 2), and aldosterone-producing cell clusters (APCCs) (e.g., specimen 1b in Figure 2). Specific tracer binding in these CYP11B2-positive areas ranged from 8.6 to 19.1 kBq/cm^2^. In specimens designated as APAs upon postsurgical visual examination (characterized by a typical golden color and dense morphology of the tissue, e.g., specimens 1a, 2a, and 3a in Figure 2), tracer binding ranged from 2.5 to 19.7 kBq/cm^2^ (*n* = 6). Notably, tracer binding was evenly distributed across APA tissue sections, in contrast to adrenal cortex sections (e.g., specimens 1b and 3b in Figure 2), for which diffuse expression patterns and “hot spots” were seen. There was no evidence of elevated tracer uptake in CYP11B2-negative specimens. In contrast, tracer binding was consistently low in specimens from control subjects (2.6 ± 1.8 kBq/cm^2^; *n* = 3) and in CYP11B2-negative areas in specimens from patients with PHA (3.2 ± 1.1 kBq/cm^2^; *n* = 4).

## Discussion

PHA is a common type of secondary hypertension, which affects as much as 1% of the population, and causes significant morbidity and premature mortality due to both hypertension and the high levels of aldosterone. Although medical treatment can prevent some of the adverse effects of PHA, in patients with unilateral disease, surgery is often more effective in reducing the blood pressure, morbidity, and risk of mortality and gives a higher quality of life. A practical, scalable, and expedient diagnosis will allow many more PHA patients to be identified for surgery and potentially cured. PET imaging of CYP11B2 expression in the adrenal glands can meet this need by identifying and lateralizing APAs noninvasively.

The parent compound of [^18^F]AldoView was reported as a benchmark for the Merck & Co. benzimidazole series of aldosterone synthase inhibitors due to its high potency and selectivity for human CYP11B2 (IC_50_ 4.7 nM vs 435 nM for *h*CYP11B1), lack of “off target” binding, high metabolic stability, and favorable pharmacokinetic profile.^19^ The presence of multiple fluorine substituents presented a convenient option for labeling with fluorine-18, to give [^18^F]AldoView, while fully retaining the chemical structure. We have recently developed sulfonium salts as a new class of leaving groups for the radiofluorination of drug-like molecules,^20,23^ and in this case, incorporation of a dibenzothiophenium salt in the 3-position of the pyridine moiety provided a practical and efficient route to the precursor for labeling. Treatment with [^18^F]fluoride allowed direct labeling in a single step in 42 ± 8% (decay-corrected; *n* = 12) radiochemical yield. The use of fluorine-18 (half-life 110 min) for labeling and the high efficiency of the labeling reaction ensure that [^18^F]AldoView can become widely available for routine diagnostic use.

The pharmacokinetic properties of [^18^F]AldoView were characterized preclinically with PET imaging and biodistribution studies in mice. PET/CT imaging in mice revealed a high initial uptake in the liver, with rapid clearance, resulting in low background in all tissues surrounding the adrenals glands at later time points. In the biodistribution studies, the liver uptake was less pronounced, whereas the peak uptake and clearance in the kidneys occurred at a later time point. These discrepancies may reflect physiological effects of the anesthesia in the animals imaged with PET.^24^ The metabolic stability of the tracer appears adequate, with 21% intact parent fraction measured in blood after 60 min. It should be noted that there are substantial species differences between adrenal enzymes and that rodents do not provide a suitable model for human CYP11B2.^25,26^ In a recent study by Novartis, the potency of aldosterone synthase inhibitors for rat CYP11B2 was shown to inversely correlate with CYP11B2/CYP11B1 selectivity for the human isoforms.^27^ The adrenal uptake and clearance profile of [^18^F]AldoView in mice, which closely resembles that of the kidneys, is therefore encouraging and consistent with the high *h*CYP11B2 selectivity (B2/B1 ratio of 93) of this ligand.

The binding of the tracer in the adrenal glands resected from patients with PHA was in good visual agreement with IHC staining for CYP11B2 and demarcated areas of intense uptake in some but not all of the designated APAs. In the cortex, diffuse binding patterns were evident, with highly localized “hot spots” consistent with APCCs. In tissue from control cases, that is, from patients with CYP11B2-negative adrenal tumors, tracer binding was consistently low. The ratio of tracer uptake between CYP11B2-positive and -negative areas ranged from 2 to 9, with >95% specific binding in areas with a high tracer uptake. Surgical removal of the adrenal glands from the PHA patients included in our study resulted in good clinical outcomes. In cases 1 and 3, where phosphorimaging with [^18^F]AldoView revealed APCCs in the adrenal cortex, adrenalectomy resulted in complete cure of the patients as evident from a normalized blood pressure as well as normal renin and aldosterone levels. The subjects 2, 4, and 5 still had elevated but controlled blood pressure postoperatively. Treatment with antihypertensive medication could be reduced from using three to one drug in patients 2 and 5, resulting in a blood pressure of 140/90 mm Hg. Patient 4 had a blood pressure of 160/89 mm Hg postoperatively, normalized aldosterone and renin values, and did not continue treatment with any of the six antihypertensive drugs taken preadrenalectomy. Taken together, the results suggest that [^18^F]AldoView can allow imaging of the CYP11B2 expression in human adrenals with PET.

### Study Limitations

Due to species differences in the human and murine adrenal enzymes, the preclinical characterization of [^18^F]AldoView in mice did not provide direct evidence that the tracer allows imaging of CYP11B2 in vivo. Although imaging in human adrenal sections was in good visual agreement with the IHC staining of CYP11B2, in vitro binding does not directly translate to in vivo imaging. In addition, we were unable to quantify the correlation of tracer binding with enzyme expression due to the lack of independent methods to measure the aldosterone synthase expression. This was particularly challenging in tissues designated as APAs by morphological inspection, for which the IHC staining of CYP11B2 gave inconclusive results due to the lack of contrast, and tracer binding varied considerably between the cases. We were also unable to demonstrate lack of tracer binding to areas with high CYP11B1 expression, as in our hands, antibody staining of this enzyme gave inconclusive results (uniform staining in tissue sections). Finally, we note that, although CYP11B2 is the only known source of aldosterone, regulation of aldosterone secretion is not entirely transcriptional, and therefore, CYP11B2 expression measured with PET may not correlate directly with the rate of aldosterone production in the adrenals.

### Translational Outlook

The study aim was to address the unmet clinical need for a noninvasive method to better diagnose PHA and to stratify patients with unilateral PHA for surgical treatment. [^18^F]AldoView, the first highly selective aldosterone synthase tracer, was developed to allow imaging of the adrenal *h*CYP11B2 expression with PET. In preclinical studies in mice, the PET tracer showed a favorable pharmacokinetic profile and organ distribution, suggesting that in vivo imaging will be feasible. Binding studies in tissue sections from surgically resected adrenals provided evidence for a highly selective tracer binding to *h*CYP11B2. The results are encouraging and warrant translation to human studies. First-in-human PET scans are due to commence in the near future. The results will determine the diagnostic efficacy of image-derived adrenal enzymatic lateralization with PET/CT.

## Experimental Section

### General Procedures and Equipment

Reagents were purchased from Sigma-Aldrich, Acros Organics, or Fluorochem and were used without further purification. Purification of compounds by flash column chromatography was performed on silica (SiO_2_ 60; 40–63 μm), followed by the evaporation of solvents in vacuo. Analytical thin-layer chromatography was carried out on silica gel 60 F254 plates with visualization by ultraviolet light, cerium ammonium molybdate, ninhydrin, or potassium permanganate dip. Petrol refers to the distillation fraction of petroleum ether with a boiling point ranging from 40 to 60 °C. Nuclear MR (NMR) spectra were recorded at room temperature. Bruker AVANCE 400 and 500 instruments were operated at a frequency of 400 or 500 MHz for ^1^H NMR and 126 MHz for ^13^C NMR, respectively. ^19^F NMR spectra were recorded on a Bruker AVANCE 300 instrument at a frequency of 282 MHz. Chemical shifts are reported in parts per million (ppm) on the delta scale, and coupling constants (*J*) are given in hertz. All spectra were internally referenced to the deuterated solvent used. Data are presented as follows: chemical shift, multiplicity (s = singlet, d = doublet, dd = doublet of doublets, ddd = doublet of doublet of doublets, t = triplet, q = quartet, quint = quintet, m = multiplet, and b = broad), coupling constant, and integration. Carbons with the same chemical shift are reported as follows: chemical shift (× carbons). NMR spectra for the compounds **3**, **4**, **6**, and **7** can be found in the Supporting Information. High-resolution mass spectra (HRMS) data were recorded on a microTOF spectrometer equipped with an orthogonal electrospray interface. The parent ions [M]^+^, [M + H]^+^, [M + K]^+^, [M + Li]^+^, or [M + Na]^+^ are reported. The purity of the intermediate compounds **1–6**, as determined by NMR, was found to be >95% in each case. The purity of compound **7**, as assessed by HPLC, was >97% (Figure S1).

### *N*-Cyclopropyl-4,5-difluoro-2-nitroaniline (**1**)

To a stirring solution of 2,4,5-trifluoronitrobenzene (5.0 g, 3.24 mL, 28.24 mmol, 1.0 equiv) and triethylamine (8.57 g, 11.8 mL, 84.70 mmol, 3.0 equiv) in tetrahydrofuran (25.7 mL) at room temperature, cyclopropylamine (6.65 g, 8.07 mL, 118 mmol, 4.2 equiv) was added dropwise over 1 h (the solution became very thick during the addition). The resulting yellow mixture was stirred at 60 °C for 2 h. After cooling to room temperature, the reaction was quenched with water (20 mL) and diluted with ethyl acetate (30 mL). The organic layer was separated, and the aqueous layer was extracted with ethyl acetate (2 × 30 mL). The combined organic layers were washed with water, brine, dried over MgSO_4_, filtered, and concentrated in vacuo. The crude product was purified by flash column chromatography (ethyl acetate/petrol 0 to 10%) on a silica gel to afford the desired product as a yellow solid (690 mg) in 11% yield. ^1^H NMR (CDCl_3_, 500 MHz): δ 8.09 (s, 1H), 8.03 (dd, *J* = 10.7, 8.3 Hz, 1H), 7.09 (dd, *J* = 12.6, 6.9 Hz, 1H), 2.54 (tdd, *J* = 6.8, 3.4, 1.8 Hz, 1H), 0.99–0.92 (m, 2H), and 0.71–0.65 (m, 2H). Consistent with literature data.^21^

### *N*-Cyclopropyl-4,5-difluorobenzene-1,2-diamine (**2**)

Under an inert atmosphere (argon), palladium on charcoal (10%; 34 mg, 0.031 mmol, 0.01 equiv) was added to a solution of *N*-cyclopropyl-4,5-difluoro-2-nitroaniline (**1**; 683 mg, 3.18 mmol, 1.0 equiv) in methanol (12.7 mL). Hydrogen gas was introduced via a balloon, and the resulting mixture was stirred at room temperature for 16 h. The reaction mixture was subsequently filtered through a pad of Celite with ethyl acetate (20 mL) and concentrated in vacuo. The crude product was purified by flash column chromatography (ethyl acetate/petrol 0 to 50%) on a silica gel to afford the desired product as a brown solid (540 mg) in 92% yield. ^1^H NMR (CDCl_3_, 500 MHz): δ 6.82 (dd, *J* = 12.5, 7.8 Hz, 1H), 6.51 (dd, *J* = 11.2, 7.6 Hz, 1H), 3.82 (s, 1H), 3.16 (s, 2H), 2.38 (tt, *J* = 6.7, 3.6 Hz, 1H), 0.79–0.71 (m, 2H), and 0.55–0.46 (m, 2H). Consistent with literature data.^21^

### 1-Cyclopropyl-5,6-difluoro-2-(5-fluoropyridin-3-yl)-1*H*-benzo[*d*]imidazole (**3**)

Oxone (326 mg, 1.06 mmol, 0.65 equiv) was added to a solution of *N*-cyclopropyl-4,5-difluorobenzene-1,2-diamine (**2**; 300 mg, 1.63 mmol, 1 equiv) and 5-fluoronicotinaldehyde (224 mg, 180 μL, 1.79 mmol, 1.1 equiv) in dimethylformamide/water (97%/3% v/v; 4.1 mL). The mixture was stirred at room temperature for 1 h. The reaction was quenched with water (20 mL) and diluted with ethyl acetate (20 mL), followed by the addition of solid K_2_CO_3_ until the aqueous layer reached pH = 9. The organic layer was separated, and the aqueous layer was extracted with ethyl acetate (3 × 30 mL). The combined organic layers were washed with water, brine, dried over MgSO_4_, filtered, and concentrated in vacuo. The crude product was purified by flash column chromatography (ethyl acetate/dichloromethane 0 to 50%) on silica gel to afford the desired product as an orange solid (298 mg) in 64% yield. ^1^H NMR (CDCl_3_, 500 MHz): δ 9.04 (t, *J* = 1.7 Hz, 1H), 8.61 (d, *J* = 2.8 Hz, 1H), 8.01 (ddd, *J* = 9.1, 2.8, 1.7 Hz, 1H), 7.57 (dd, *J* = 10.3, 7.3 Hz, 1H), 7.41 (dd, *J* = 9.7, 7.0 Hz, 1H), 3.58 (tt, *J* = 6.8, 3.8 Hz, 1H), 1.25 (dq, *J* = 6.9, 1.2 Hz, 2H), and 0.84–0.79 (m, 2H); ^13^C NMR (CDCl_3_, 126 MHz): δ 159.1 (d, *J* = 258.2 Hz), 151.1, 150.2 (dd, *J* = 23.9, 15.4 Hz), 146.9 (dd, *J* = 21.4, 15.4 Hz), 145.5 (d, *J* = 4.3 Hz), 139.2 (d, *J* = 23.1 Hz), 137.8 (d, *J* = 11.0 Hz), 132.6 (d, *J* = 10.8 Hz), 127.8 (d, *J* = 4.2 Hz), 123.2 (d, *J* = 20.0 Hz), 107.6 (d, *J* = 19.9 Hz), 99.2 (d, *J* = 23.1 Hz), 26.6, and 9.1 (2C); ^19^F NMR (CDCl_3_, 282 MHz): δ −125.72, −139.48 (d, *J* = 20.6 Hz) and −142.30 (d, *J* = 20.6 Hz); and HRMS: 290.0901 (C_15_H_10_F_3_N_3_+H^+^) calcd, 290.0900.

### 2-(5-Bromopyridin-3-yl)-1-cyclopropyl-5,6-difluoro-1*H*-benzo[*d*]imidazole (**4**)

Oxone (252 mg, 0.82 mmol, 0.65 equiv) was added to a solution of *N*-cyclopropyl-4,5-difluorobenzene-1,2-diamine (**2**; 232 mg, 1.26 mmol, 1 equiv) and 5-bromonicotinaldehyde (258 mg, 1.39 mmol, 1.1 equiv) in dimethylformamide/water (97%/3% v/v; 3.1 mL). The mixture was stirred at room temperature for 1 h. The reaction was quenched with water (20 mL) and diluted with ethyl acetate (20 mL), followed by the addition of solid K_2_CO_3_ until the aqueous layer reached pH = 9. The organic layer was separated and the aqueous layer was extracted with ethyl acetate (3 × 30 mL). The combined organic layers were washed with water, brine, dried over MgSO_4_, filtered, and concentrated in vacuo. The crude product was purified by flash column chromatography (ethyl acetate/dichloromethane 0 to 30%) on silica gel to afford the desired product as a brown solid (324 mg) in 73% yield. ^1^H NMR (CDCl_3_, 500 MHz): δ 9.13 (d, *J* = 1.9 Hz, 1H), 8.80 (d, *J* = 2.2 Hz, 1H), 8.45 (t, *J* = 2.1 Hz, 1H), 7.57 (dd, *J* = 10.2, 7.3 Hz, 1H), 7.41 (dd, *J* = 9.7, 7.0 Hz, 1H), 3.57 (tt, *J* = 6.9, 3.8 Hz, 1H), 1.29–1.21 (m, 2H), and 0.85–0.78 (m, 2H); ^13^C NMR (CDCl_3_, 126 MHz): δ 151.8, 151.0, 149.6 (dd, *J* = 40.2, 15.2 Hz), 147.7, 147.7 (dd, *J* = 38.9, 17.6 Hz), 138.9, 138.0 (d, *J* = 24.0 Hz), 132.7 (d, *J* = 10.8 Hz), 128.0, 120.8, 107.7 (d, *J* = 19.7 Hz), 99.2 (d, *J* = 23.2 Hz), and 26.6, 9.2 (2C); ^19^F NMR (CDCl_3_, 282 MHz): δ −139.41 (d, *J* = 20.5 Hz) and −142.23 (d, *J* = 20.5 Hz); and HRMS: 350.0109 (C_15_H_10_BrF_2_N_3_+H^+^) calcd, 350.0104.

### 2-Ethylhexyl 3-((3′,5′-Dimethoxy-5-methyl-[1,1′-biphenyl]-2-yl)thio)propanoate (**5**)

The compound was prepared as previously reported.^20^ In brief, heating of a mixture of 2-bromo-1-iodo-4-methylbenzene (1 equiv), 2-ethylhexyl 3-mercaptopropanoate (1 equiv), triethylamine (2 equiv), tris(dibenzylideneacetone)-dipalladium(0) (1.5 mol %), and Xantphos (3 mol %) in toluene at 125 °C for 2 h and subsequent workup by filtration over Celite and flash chromatography provided 2-ethylhexyl 3-((2-bromo-4-methylphenyl)thio)propanoate as a colorless oil (96%). This intermediate (1 equiv) was further reacted in a Suzuki cross-coupling by refluxing it with (3,5-dimethoxy-phenyl)boronic acid (1.25 equiv), potassium carbonate (4 equiv), and PEPPSI-IPr catalyst (3 mol %) in a mixture of toluene and water for 2 h. Filtration over Celite, followed by an aqueous workup and flash column chromatography afforded the title compound as a colorless oil (95%).

### 1-Cyclopropyl-2-(5-((3′,5′-dimethoxy-5-methyl-[1,1′-biphenyl]-2-yl)thio)pyridin-3-yl)-5,6-difluoro-1*H*-benzo[*d*]imidazole (**6**)

To a flame-dried three-necked round-bottom flask equipped with an argon inlet and condenser were added sequentially tris(dibenzylideneacetone)dipalladium(0) (168 mg, 0.19 mmol, 0.2 equiv), bis(2-diphenylphosphinophenyl)ether (198 mg, 0.37 mmol, 0.4 equiv), 2-(5-bromopyridin-3-yl)-1-cyclopropyl-5,6-difluoro-1*H*-benzo[*d*]imidazole (**4**; 322 mg, 0.92 mmol, 1 equiv), and toluene (4.8 mL). The resulting mixture was stirred at room temperature for 10 min. A solution of 2-ethylhexyl 3-((3′,5′-dimethoxy-5-methyl-[1,1′-biphenyl]-2-yl)thio)propanoate (**5**; 448 mg, 1.01 mmol, 1.1 equiv) in toluene (4.8 mL) was subsequently added, followed by the addition of potassium *tert*-butoxide (144 mg, 1.28 mmol, 1.4 equiv). The reaction mixture was degassed for 5 min using a balloon filled with argon and a vent needle and then heated at 125 °C for 2 h. After cooling to room temperature, the reaction mixture was concentrated in vacuo. The crude product was purified by flash column chromatography (ethyl acetate/petrol 10 to 60%) on silica gel to afford the desired product as an orange solid (457 mg) in 94% yield. ^1^H NMR (CDCl_3_, 500 MHz): δ 8.89 (d, *J* = 1.9 Hz, 1H), 8.38 (d, *J* = 2.1 Hz, 1H), 7.92 (t, *J* = 2.1 Hz, 1H), 7.52 (dd, *J* = 10.3, 7.3 Hz, 1H), 7.42–7.29 (m, 2H), 7.20 (d, *J* = 1.9 Hz, 1H), 7.17–7.11 (m, 1H), 6.45 (d, *J* = 2.2 Hz, 2H), 6.39 (t, *J* = 2.3 Hz, 1H), 3.72 (s, 6H), 3.39 (tt, *J* = 7.0, 3.8 Hz, 1H), 2.37 (s, 3H), 1.15–1.04 (m, 2H), and 0.92–0.80 (m, 2H); ^13^C NMR (CDCl_3_, 126 MHz): δ 160.4, 151.9 (d, *J* = 3.3 Hz), 150.7, 149.4 (dd, *J* = 32.5, 15.3 Hz), 147.5 (dd, *J* = 30.0, 15.4 Hz), 147.0, 145.1, 142.5, 139.1, 137.7 (d, *J* = 10.6 Hz), 136.9, 135.1, 134.3, 132.5 (d, *J* = 10.7 Hz), 131.9, 129.5, 129.4, 127.7, 126.5, 107.6 (2C), 107.5 (d, *J* = 19.8 Hz), 99.7, 99.0 (d, *J* = 23.1 Hz), 55.5 (2C), 26.5, 21.2, and 9.0 (2C); ^19^F NMR (CDCl_3_, 282 MHz): δ −140.01 (d, *J* = 20.6 Hz) and −142.67 (d, *J* = 20.6 Hz); and HRMS: 530.1709 (C_30_H_25_F_2_N_3_O_2_S+H^+^) calcd, 530.1714.

### 5-(5-(1-Cyclopropyl-5,6-difluoro-1*H*-benzo[*d*]imidazol-2-yl)pyridin-3-yl)-2,4-dimethoxy-8-methyl-5*H*-dibenzo[*b*,*d*]thiophen-5-ium Trifluoromethanesulfonate (**7**)

To a solution of 1-cyclopropyl-2-(5-((3′,5′-dimethoxy-5-methyl-[1,1′-biphenyl]-2-yl)thio)pyridin-3-yl)-5,6-difluoro-1*H*-benzo[*d*]imidazole (**6**; 280 mg, 0.53 mmol, 1 equiv) and sodium trifluoromethanesulfonate (182 mg, 1.05 mmol, 2 equiv) in acetone (analytical reagent grade; 3.4 mL) at 0 °C, was added a freshly prepared solution of Ca(OCl)_2_ (a fresh bottle of technical grade reagent (65% w/w) was used; 45.4 mg, 0.21 mmol, 0.4 equiv) in an aqueous acetate buffer (1 M, pH = 4; 3.4 mL). Under stirring, the resulting yellow solution was allowed to come to room temperature. Stirring was then continued for 1 h. The reaction was subsequently diluted with dichloromethane (30 mL) and water (20 mL). The organic layer was separated, and the aqueous layer was extracted with dichloromethane (2 × 30 mL). The combined organic layers were washed with an aqueous solution of sodium trifluoromethanesulfonate (1 M; 10 mL), dried over MgSO_4_, filtered, and concentrated in vacuo. The crude product was purified by flash column chromatography on silica gel. Initially, an ethyl acetate/petrol gradient from 30 to 70% was used to remove lipophilic impurities. The column was then continued using a methanol/dichloromethane gradient from 1 to 3% to remove more polar impurities before eluting the product fraction at 3% methanol/dichloromethane. The title compound was isolated as a yellow solid (114 mg) in 32% yield (48 mg of the starting material was recovered). ^1^H NMR (CD_3_CN, 400 MHz): δ 9.39 (d, *J* = 1.9 Hz, 1H), 9.09 (d, *J* = 2.3 Hz, 1H), 8.20 (dt, *J* = 1.8, 0.7 Hz, 1H), 8.07–8.02 (m, 2H), 7.60–7.50 (m, 3H), 7.45 (d, *J* = 2.1 Hz, 1H), 6.78 (d, *J* = 2.1 Hz, 1H), 4.00 (s, 3H), 3.94 (s, 3H), 3.34 (tt, *J* = 6.9, 3.8 Hz, 1H), 2.57 (d, *J* = 0.7 Hz, 3H), 0.92–0.78 (m, 2H), and 0.65–0.52 (m, 2H); ^13^C NMR (CD_3_CN, 126 MHz): δ 169.1, 159.8, 155.7, 152.1, 151.21, 150.4 (dd, *J* = 32.2, 15.7 Hz), 147.7 (dd, *J* = 31.1, 15.3 Hz), 147.5, 143.3, 140.8, 138.6 (d, *J* = 10.7 Hz), 136.8, 133.9 (d, *J* = 11.4 Hz), 133.9, 129.8, 129.1, 129.0, 126.7, 125.8, 107.8 (d, *J* = 19.6 Hz), 107.3, 103.3, 101.6, 100.6 (d, *J* = 23.3 Hz), 58.2, 57.7, 27.0, 21.8, and 9.0 (2C); ^19^F NMR (CD_3_CN, 282 MHz): δ −79.3, −142.07 (d, *J* = 20.0 Hz) and −144.97 (d, *J* = 20.0 Hz); and HRMS: 528.1555 (C_30_H_24_F_2_N_3_O_2_S^+^) calcd, 528.1552.

### Radiosynthesis of [^18^F]AldoView

All labeling reactions were performed manually using [^18^F]fluoride in [^18^O]water. Radio-HPLC was performed on an Agilent 1200 HPLC system equipped with a 1200 Series Diode Array Detector and a GABI Star NaI(Tl) scintillation detector. The system was used for the purification of [^18^F]AldoView, quality control, and for metabolite analysis.

Cartridges for solid-phase extraction were purchased from Waters and conditioned on the day of the labeling experiment as follows:

- Sep-Pak Accell Plus QMA Light (130 mg, WAT023525): aqueous sodium hydroxide (1 M, 5 mL), HPLC water (10 mL), aqueous potassium carbonate (1 M, 1 mL), HPLC water (10 mL), and air (10 mL)
- Sep-Pak Alumina N Plus Light: (280 mg, WAT023561): HPLC water (1 mL) and air (10 mL)
- Sep-Pak C-18 Plus Light (130 mg, WAT023501): methanol (5 mL), HPLC water (10 mL), and air (10 mL)

Radiolabeling of [^18^F]AldoView was carried out as described previously^20,23^ using the dibenzothiophene sulfonium salt labeling precursor **7**. [^18^F]Fluoride in [^18^O]water (2 GBq) was trapped on a Sep-Pak QMA Light cartridge and released with a solution of Kryptofix 222 (30 mM) and potassium bicarbonate (30 mM) in acetonitrile and water (85%/15% v/v; 0.5 mL). The solvent was removed by heating at 90 °C under a stream of nitrogen, and [^18^F]fluoride was dried by azeotropic distillation with acetonitrile (2 × 0.5 mL; 90 °C). The reaction vial was subsequently capped, a solution of the sulfonium salt **7** (2 mg) in dimethylsulfoxide (0.5 mL) was added, and the mixture was stirred at 110 °C for 15 min. After cooling, water (1 mL) was added and the reaction mixture (Figure S2A) was purified by radio-HPLC using a Phenomenex Luna C-18(2) column (5 μm; 250 × 10 mm) at room temperature. The mobile phase consisted of water and methanol (59%/41% v/v, each containing 0.5% trifluoroacetic acid), and isocratic elution at a flow rate of 5 mL/min allowed for the isolation of the radioactive product. The fraction containing [^18^F]AldoView was diluted with water to a final volume of 20 mL and filtered over a Sep-Pak Alumina N Plus Light cartridge. The radioactive product was subsequently trapped on a Sep-Pak C-18 Plus Light cartridge, eluted with ethanol (0.5 mL), formulated in saline containing ethanol (5%), and sterilized by filtration. [^18^F]AldoView was obtained in 42 ± 8% (decay-corrected; *n* = 12) radiochemical yield, with a radiochemical purity of >99%, and with a molar activity of 31 ± 3 GBq/μmol (*n* = 4) when starting with 2 GBq of [^18^F]fluoride.

Quality control was performed on an Agilent Eclipse Plus C-18 column (5 μm; 150 × 4.6 mm) at a flow rate of 1.8 mL/min using water and methanol (each containing 0.1% trifluoroacetic acid). Gradient elution started with 35% methanol content that was increased to 70% over 7 min. The retention time was approximately 6.5 min (Figure S2B).

### Animals

All animal work was performed in compliance with the United Kingdom Home Office’s Animals (Scientific Procedures) Act 1986 and with approval of the University College London (UCL) Animal Ethics Committee. Female wild-type BALB/c mice (Charles River Laboratories, Margate, UK) were allowed to acclimatize for at least 1 week at the animal facilities at the UCL Centre for Advanced Biomedical Imaging, and they were given food and water ad libitum. When used for experiments, they were 8 to 11 weeks old and weighing approximately 20 g.

### PET/CT Imaging

Dynamic PET imaging was performed using a nanoScan PET-CT system manufactured by Mediso (Medical Imaging Systems, Budapest, Hungary). Mice (*n* = 6) were anaesthetized with isoflurane (2% v/v in oxygen) and placed on the preheated bed of the scanner (set at 38 °C). [^18^F]AldoView (7.3 ± 4.1 MBq, formulated in 100–150 μl saline containing 5% ethanol) was injected into the tail vein via intravenous cannulation. After injection, the catheter was carefully removed. The breathing rate and body temperature of the animals were closely monitored during the dynamic PET scans and, if necessary, the isoflurane dose was adjusted. Scans were recorded over 1 h, and the animals were subsequently sacrificed by cervical dislocation. Quantification of the tissue uptake (in % ID/g) was carried out based on the regions of interest manually drawn on the CT using the software package VivoQuant 1.23 (inviCRO, Boston, USA).

### Biodistribution Studies

[^18^F]AldoView (6.6 ± 1.9 MBq, formulated in saline containing 5% ethanol) was administered intravenously into the tail vein of female wild-type BALB/c mice (*n* = 3–4 per time point) without anesthesia. At designated time points (15, 30, 60, and 120 min postinjection), animals were anesthetized with isoflurane (4% v/v in oxygen) and the blood was taken by cardiac puncture. Mice were subsequently sacrificed by cervical dislocation. The organs of interest were sampled and weighed, and the radioactivity content was measured by automated gamma counting (PerkinElmer Wizard^2^). Results were expressed as % ID/g bodyweight.

### Metabolite Analysis

Blood metabolite analysis was performed as part of the biodistribution studies (*n* = 2–3 per time point). Blood samples were collected in heparin-coated tubes, and an aliquot (20 μL) was taken for gamma counting. After centrifugation (5 min, 13,000 rpm), the plasma (∼500 μL) was separated. Plasma proteins were subsequently precipitated with ice-cold acetonitrile (1 mL), and samples were centrifuged (5 min, 13,000 rpm). An aliquot of the supernatant (500 μL) was separated from the pellet and diluted with water (500 μL). The recovery of radioactivity in the supernatant was near quantitative (>95%). Analysis was carried out by radio-HPLC using a Phenomenex Onyx monolithic C-18 column (100 × 10 mm). The mobile phase consisted of water and methanol, and the flow rate was 5 mL/min. An isocratic eluent (10% methanol in water) was used for 3 min, followed by a gradient from 10 to 70% methanol over 7 min.

### Adrenal Tissue

Adrenal surgical specimens were obtained from patients with Conn’s disease, Cushing’s disease, pheochromocytoma, and adrenal tumors, who had consented to the use of their tissue for research applications. Ethical approval was obtained from the UK Multicentre Research Ethics Committee (06/Q0104/133). Following routine adrenalectomy, surgical specimens were immediately flash frozen using a slurry of isopentane and dry ice. Cryosections (20 μm) obtained from the frozen tissue blocks were mounted on lysine-coated microscope slides and stored at −80 °C.

### Quantitative Phosphorimaging

Thawed tissue sections on microscopic slides were dipped in pentane for 5 s to degrease the tissue. This step was required to avoid nonspecific binding (NSB), in particular to medullar fat. Tissue sections were subsequently rehydrated in tris-buffered saline (TBS; pH = 7.5) for 30 min and subsequently incubated with a solution of [^18^F]AldoView in TBS for 60 min (1 mL per slide). For determination of the total binding (TB), [^18^F]AldoView was diluted with TBS to a concentration of 2 MBq/mL. For the determination of NSB, a solution of the nonradioactive reference compound in ethanol (3.5 mM) was added to a solution of [^18^F]AldoView in TBS and diluted to give a final AldoView concentration of 25 μM and a final [^18^F]AldoView concentration of 2 MBq/mL. After incubation, unbound [^18^F]AldoView was removed by washing the sections in ice-cold TBS (three times for 5 min) and water (1 min). Internal standards were prepared by serial dilution of the [^18^F]AldoView solution used for determination of the TB. Tissue sections and internal standards absorbed on filter paper were left to air-dry and subsequently exposed to a phosphor screen (BAS-IP MS; GE Healthcare) overnight. Phosphorimaging was performed on a Typhoon Trio scanner (GE Healthcare). All phosphorimaging experiments were performed in at least three adjacent tissue sections per case, tissue block, and experimental condition (TB, NSB). Quantification of phosphor images was performed using the analysis software ImageJ (version 1.48).^28^ Determination of TB and NSB (in kBq/cm^2^; Table S1) was based on correlation curves generated from the internal standards. The specific [^18^F]AldoView binding was calculated as the difference between TB and NSB.

### Immunohistochemistry

IHC staining was carried out in a flash-frozen tissue on sequential sections to those used for quantitative phosphorimaging. Tissue sections were incubated with mouse monoclonal antihuman CYP11B2 (gifted by Prof. Celso E. Gomez-Sanchez; 1:1000, overnight, 4 °C), followed by biotinylated antimouse IgG (Vector Laboratories, 1:400, 1 h, room temperature) and VectaStain ABC Kit (Vector Laboratories, 30 min, room temperature). Color was developed with 3,3′-diaminobenzidine and hydrogen peroxide. Counterstaining was carried out using Mayer’s haematoxylin.

## Supporting Information Available

NMR spectra of compounds **3**, **4**, **6**, and **7**; HPLC trace of the [^18^F]AldoView labeling precursor; radio-HPLC trace of [^18^F]AldoView; [^18^F]AldoView binding in adrenal surgical specimens; and quantitative phosphorimaging with [^18^F]AldoView in tissue sections from surgically resected adrenal glands (PDF)
Molecular formula strings (CSV)

## Abbreviations

APA: aldosterone-producing adenoma
APCC: aldosterone-producing cell cluster
AVS: adrenal vein sampling
CT: computed tomography
(*h*)CYP11B1: (*human*) gene encoding 11-β hydroxylase
(*h*)CYP11B2: (*human*) gene encoding aldosterone synthase
IHC: immunohistochemistry
MRI: magnetic resonance imaging
PET: positron emission tomography
PHA: primary hyperaldosteronism
