# Supplementary material for: Development of [18F]AldoView as the First Highly Selective Aldosterone Synthase PET Tracer for Imaging of Primary Hyperaldosteronism
Source: J Med Chem. 2021 Jun 17;64(13):9321–9. doi: 10.1021/acs.jmedchem.1c00539 (PMC8273890; doi:10.1021/acs.jmedchem.1c00539)
Supplement: Supplementary file 1 — jm1c00539_si_001.pdf [file jm1c00539_si_001.pdf]

Supporting Information

for

Development of [^{18}F]AldoView as the first highly selective aldosterone synthase PET tracer for imaging of primary hyperaldosteronism

Kerstin Sander,[†] Thibault Gendron,[†] Klaudia A. Cybulska,[†] Fatih Sirindil,[†] Junhua Zhou,[‡] Tammy L. Kalber,[§] Mark F. Lythgoe,[§] Tom R. Kurzawinski,[||] Morris J. Brown,[‡] Bryan Williams,[||,#] Erik Årstad[†,*]

[†] Centre for Radiopharmaceutical Chemistry, University College London, 5 Gower Place, London WC1E 6BS, UK

[‡] William Harvey Research Institute, Barts & The London School of Medicine & Dentistry, Queen Mary University of London, Charterhouse Square, London EC1M 6BQ, UK

[§] Centre for Advanced Biomedical Imaging, University College London, 72 Huntley Street, London WC1E 6DD, UK

[||] NIHR University College London Hospitals Biomedical Research Centre, 149 Tottenham Court Road, London W1T 7DN, UK

[#] Institute of Cardiovascular Sciences, University College London, Gower Street, London WC1E 6BT, UK

* Email: e.arstad@ucl.ac.uk

Table of contents

Data file S1. ^{1}H, ^{13}C and ^{19}F NMR spectra of compounds **3**, **4**, **6** and **7** **S2**

Figure S1. HPLC trace of [^{18}F]AldoView labelling precursor **7** …..…………………………….. **S8**

Figure S2. Radio-HPLC trace of [^{18}F]AldoView ……..………………………………………... **S8**

Table S1. [^{18}F]AldoView binding in adrenal surgical specimens ……………………………… **S9**

Figure S3. Quantitative phosphorimaging with [^{18}F]AldoView in tissue sections from surgically resected adrenal glands …………………………………………………………….. **S10**

Data file S1. ^{1}H, ^{13}C and ^{19}F NMR spectra of compounds 3, 4, 6 and 7

1-Cyclopropyl-5,6-difluoro-2-(5-fluoropyridin-3-yl)-1*H*-benzo[*d*]imidazole (3)

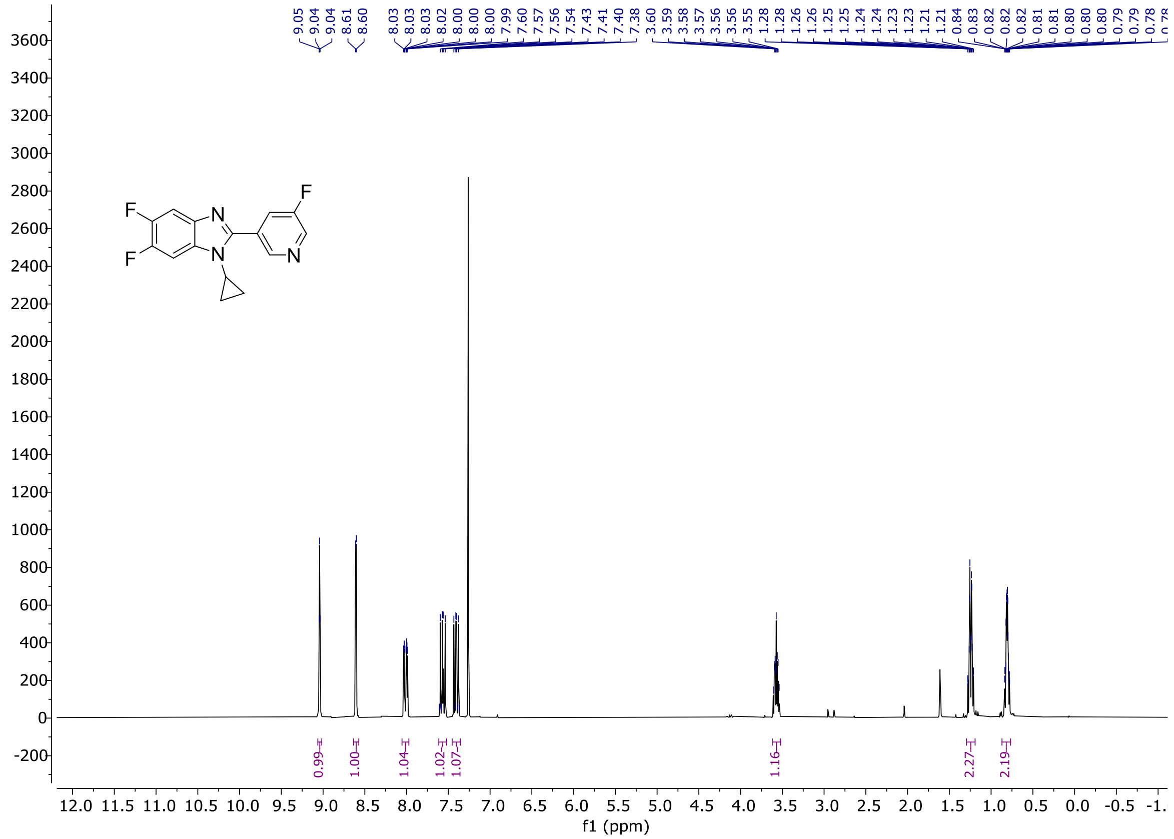

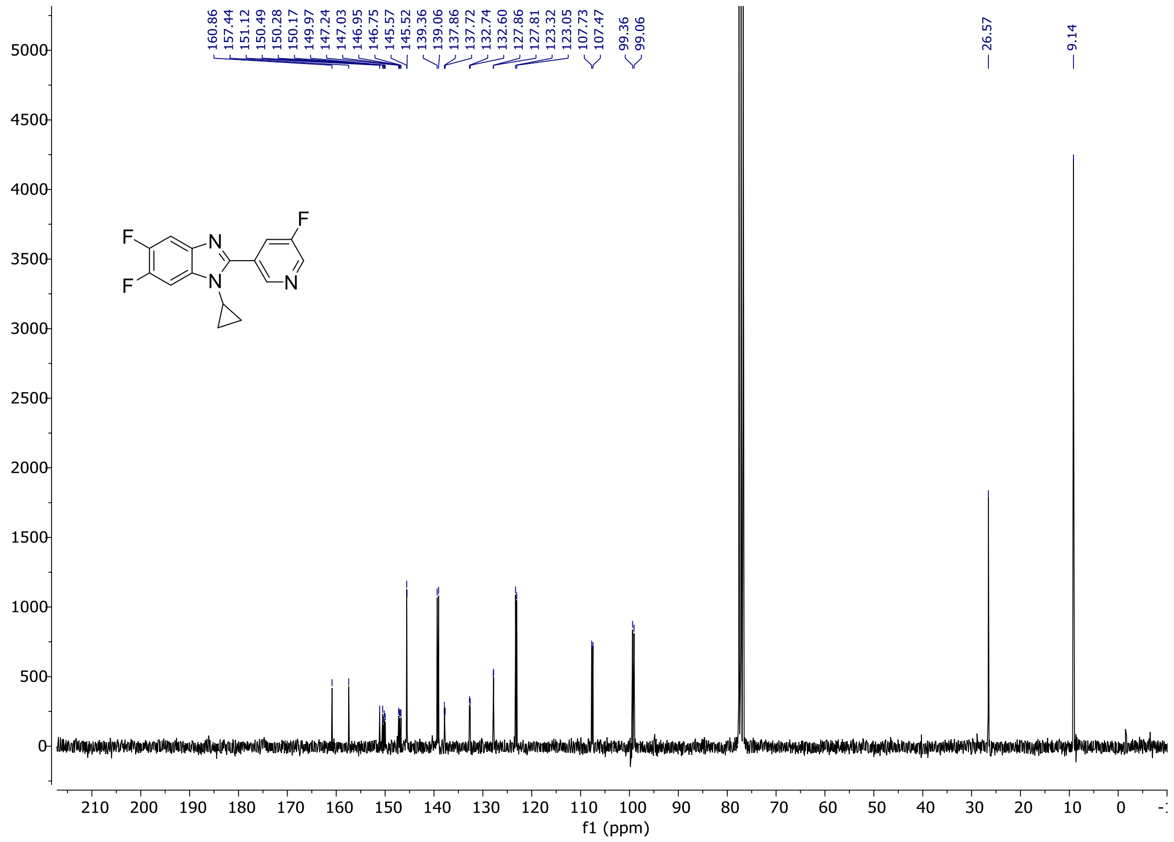

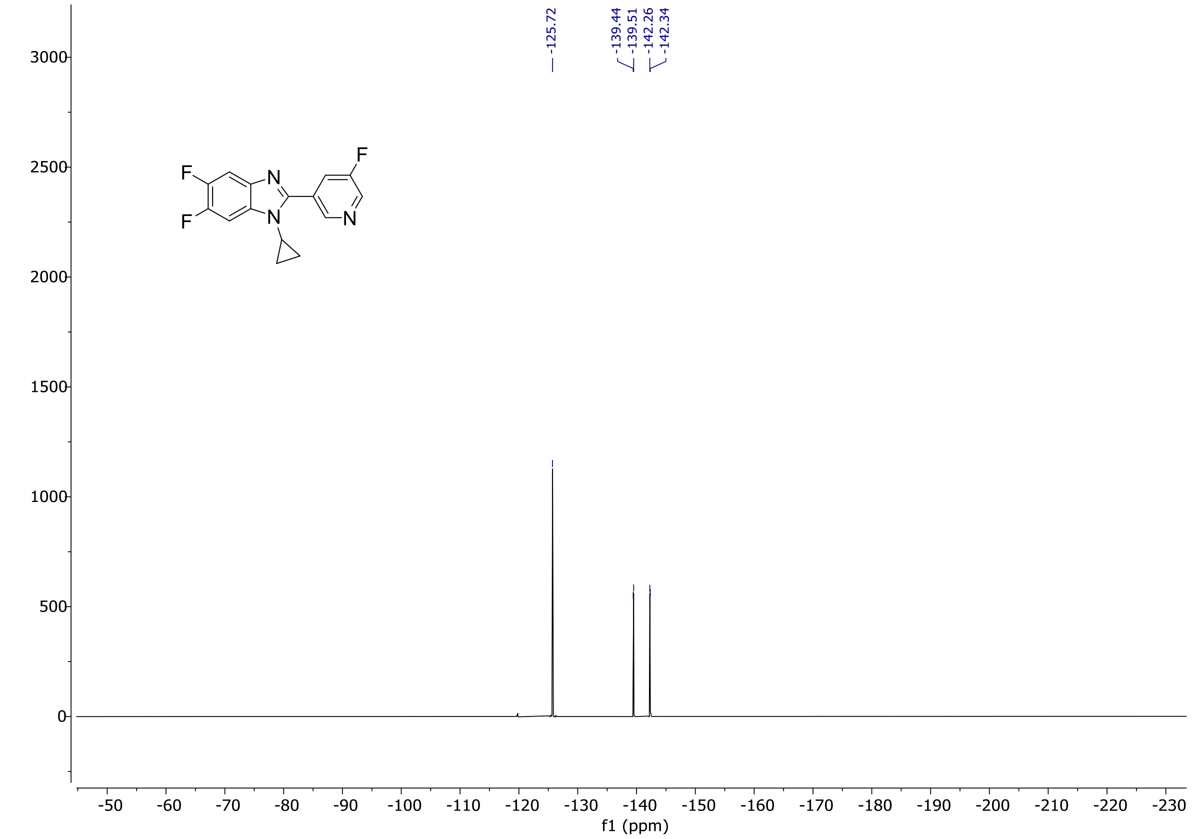

2-(5-Bromopyridin-3-yl)-1-cyclopropyl-5,6-difluoro-1*H*-benzo[*d*]imidazole (4)

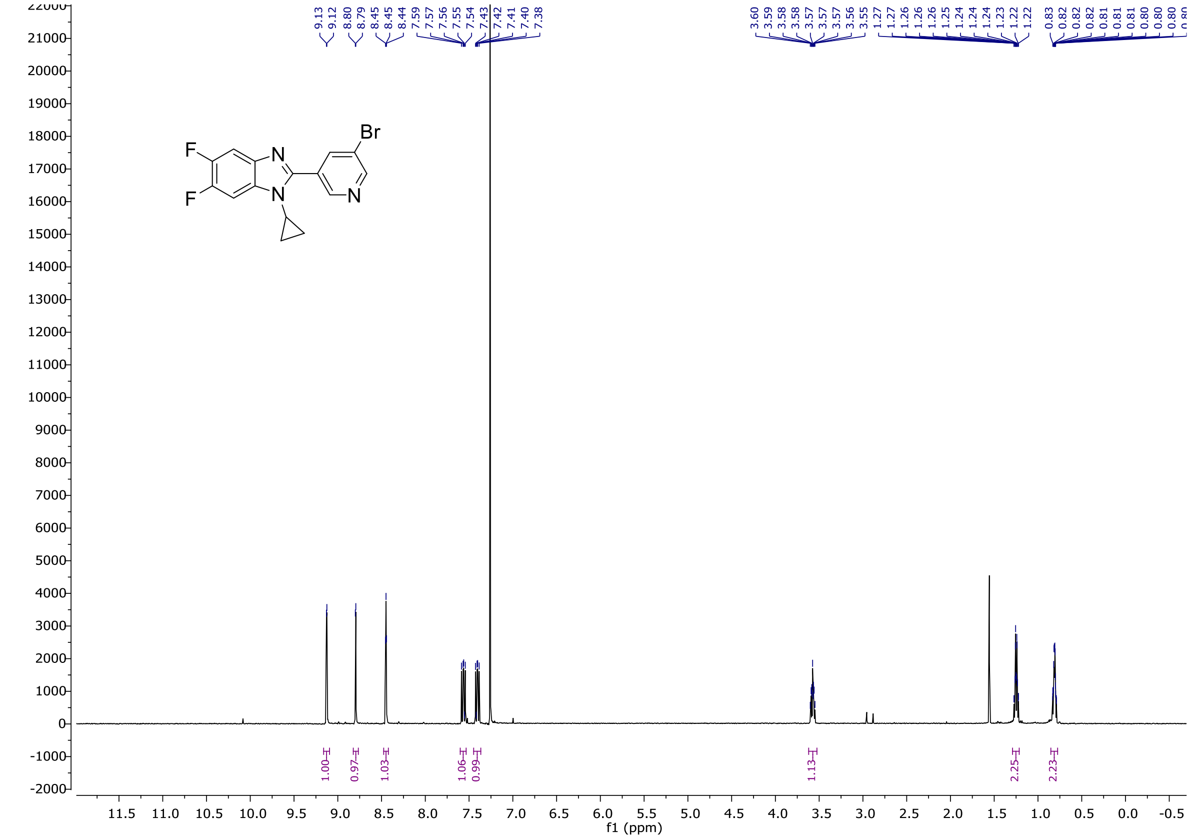

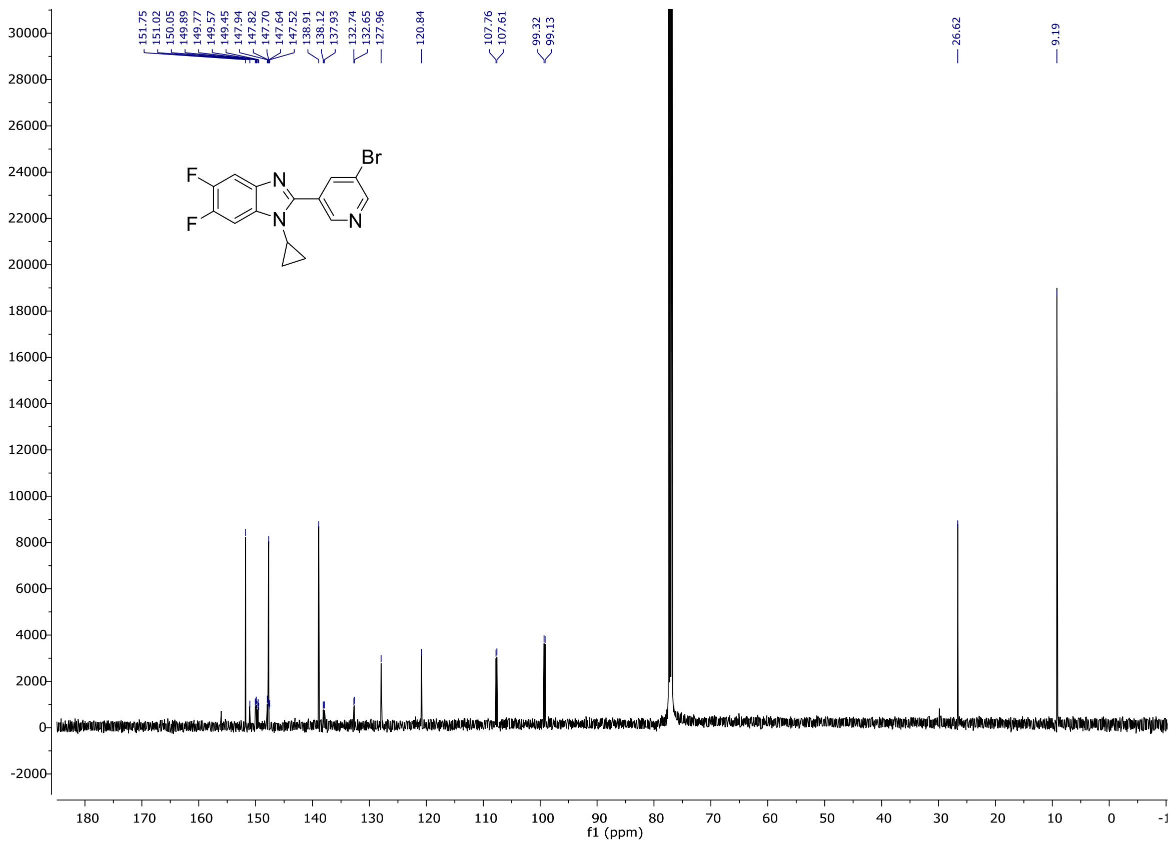
151.75
151.02
150.05
149.89
149.77
149.57
149.45
147.94
147.82
147.70
147.64
147.52
138.91
138.12
137.93
132.74
132.65
127.96
120.84
107.76
107.61
99.32
99.13
26.62
9.19
Br
F
F
N
N
N
f1 (ppm)

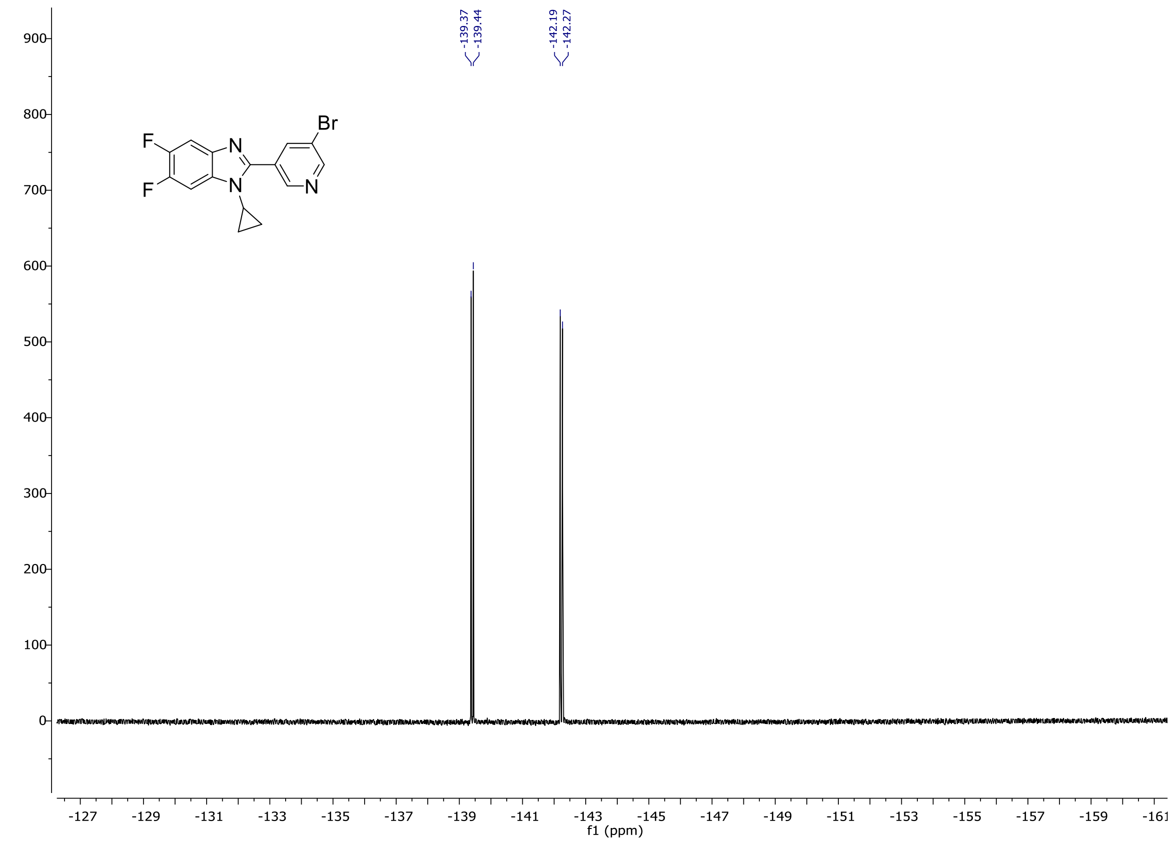
-139.37
-139.44
-142.19
-142.27
Br
F
F
N
N
N
f1 (ppm)

1-Cyclopropyl-2-(5-((3',5'-dimethoxy-5-methyl-[1,1'-biphenyl]-2-yl)thio)pyridin-3-yl)-5,6-difluoro-1*H*-benzo[*d*]imidazole (6)

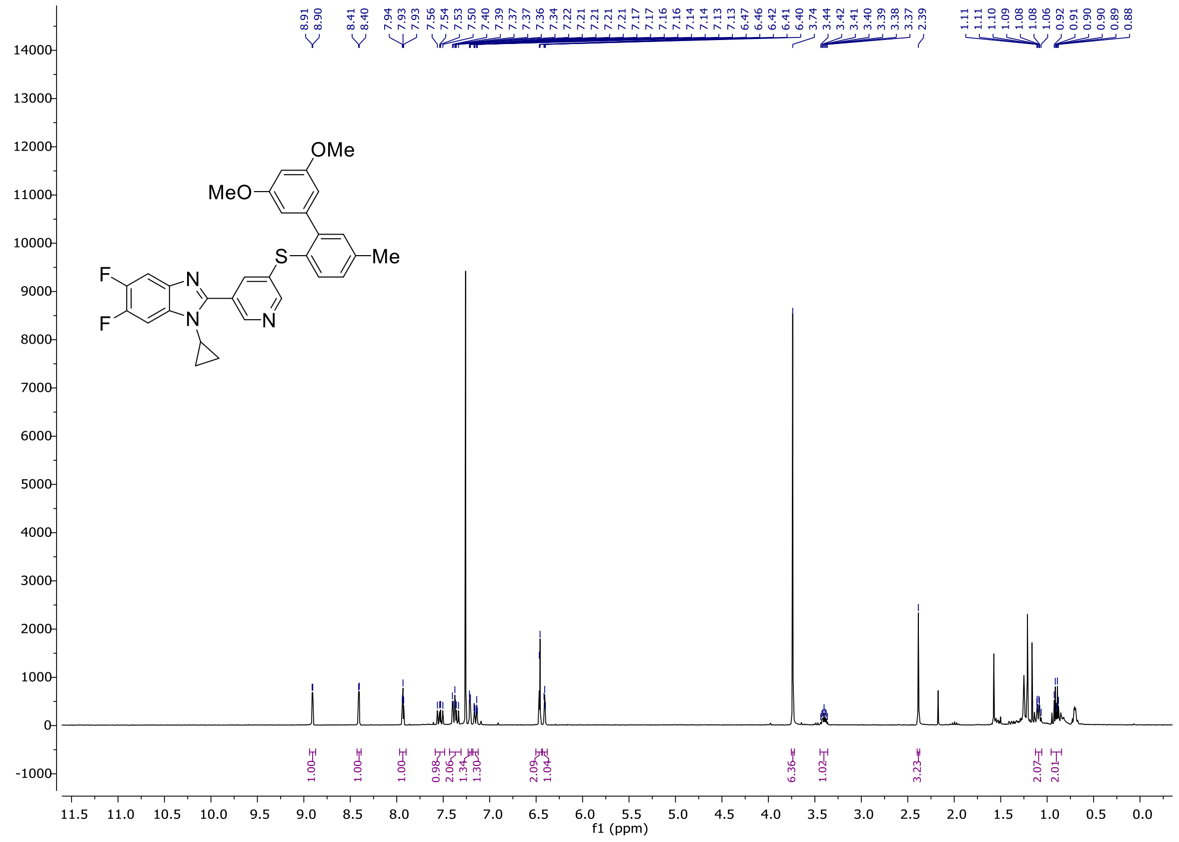

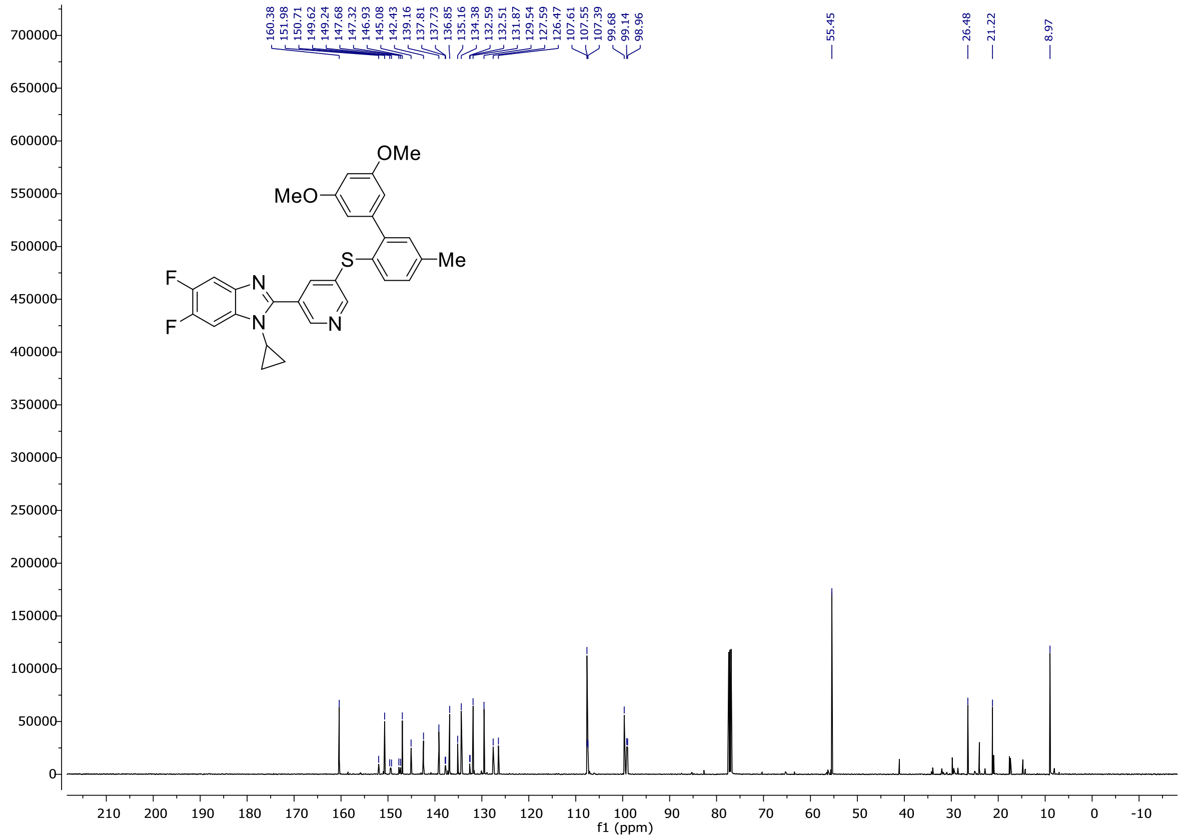

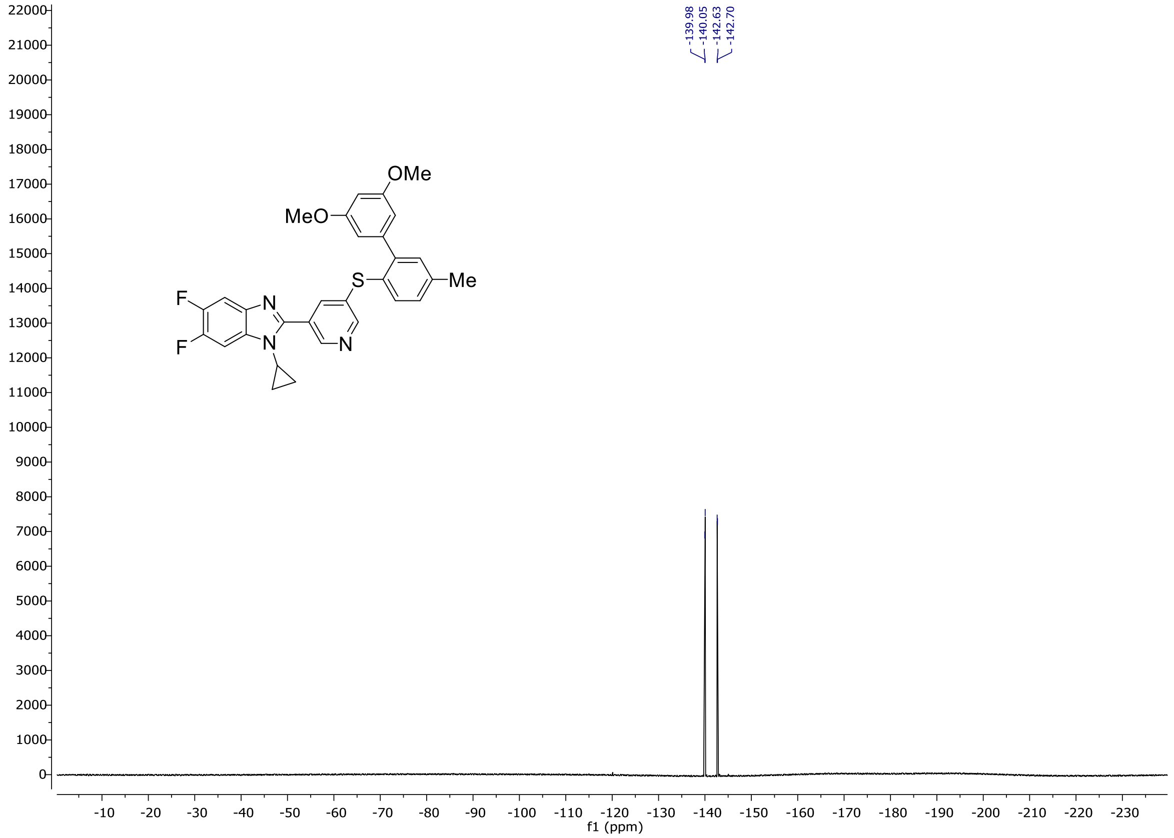

5-(5-(1-Cyclopropyl-5,6-difluoro-1*H*-benzo[*d*]imidazol-2-yl)pyridin-3-yl)-2,4-dimethoxy-8-methyl-5*H*-dibenzo[*b,d*]thiophen-5-ium trifluoromethanesulfonate (7)

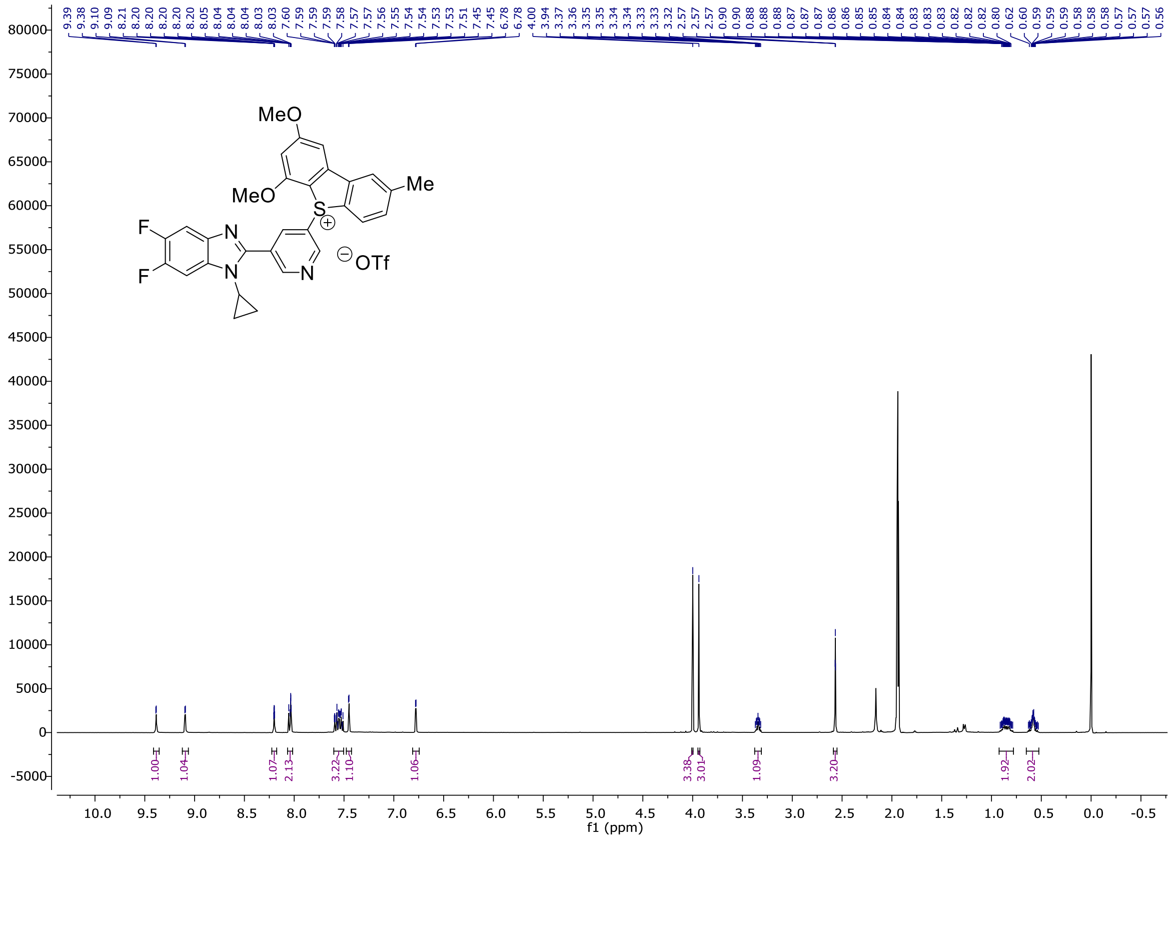

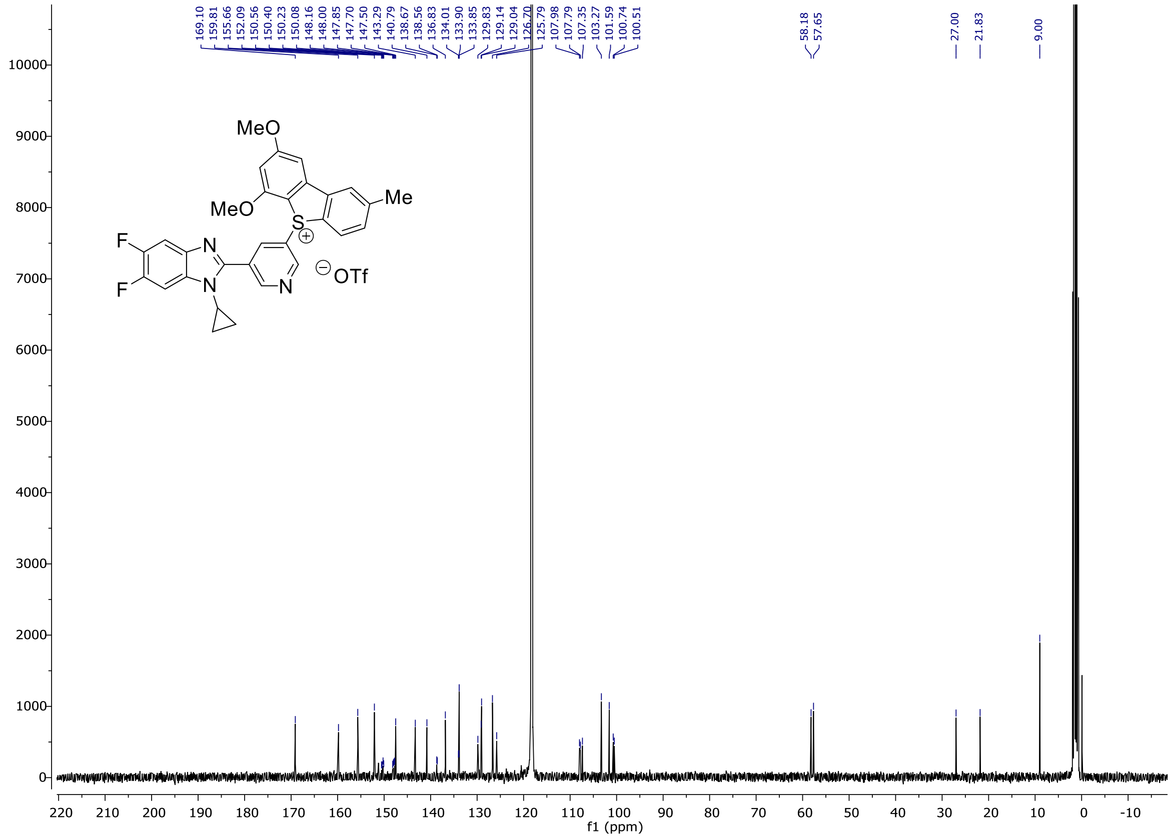
169.10
159.81
155.66
152.09
150.56
150.40
150.23
150.08
148.16
148.00
147.85
147.70
147.50
143.29
140.79
138.67
138.56
136.83
134.01
133.90
133.85
129.83
129.14
129.04
126.70
125.79
107.98
107.79
107.35
103.27
101.59
100.74
100.51
58.18
57.65
27.00
21.83
9.00
MeO
MeO
Me
S
N
N
N
F
F
OTf
f1 (ppm)

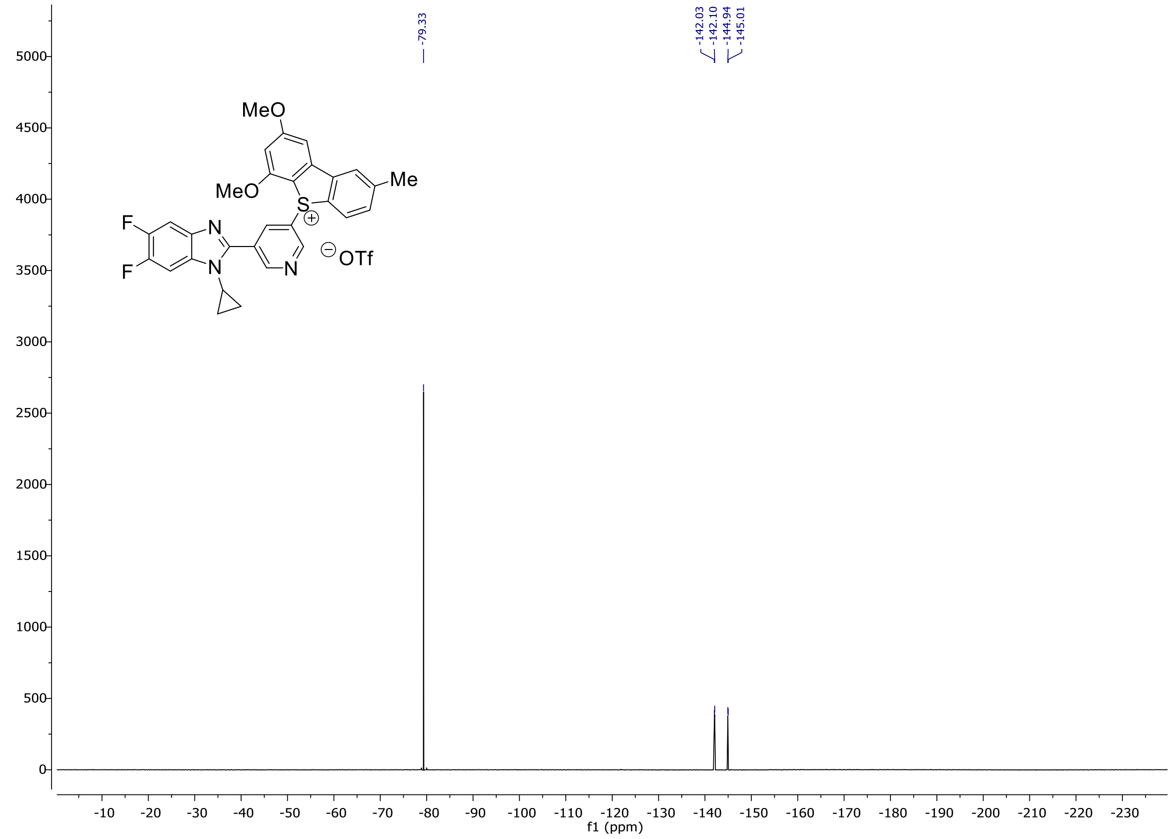
-79.33
-142.03
-142.10
-144.94
-145.01
MeO
MeO
Me
S
N
N
N
F
F
OTf
f1 (ppm)

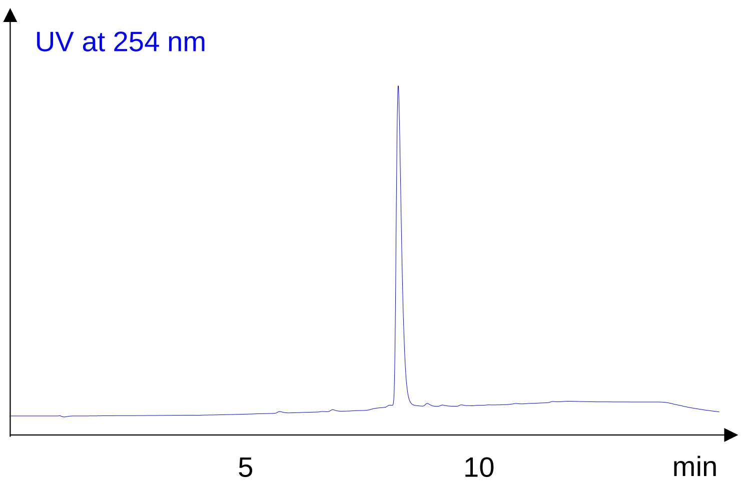

Figure S1. HPLC trace of [^{18}F]AldoView labelling precursor (compound 7)

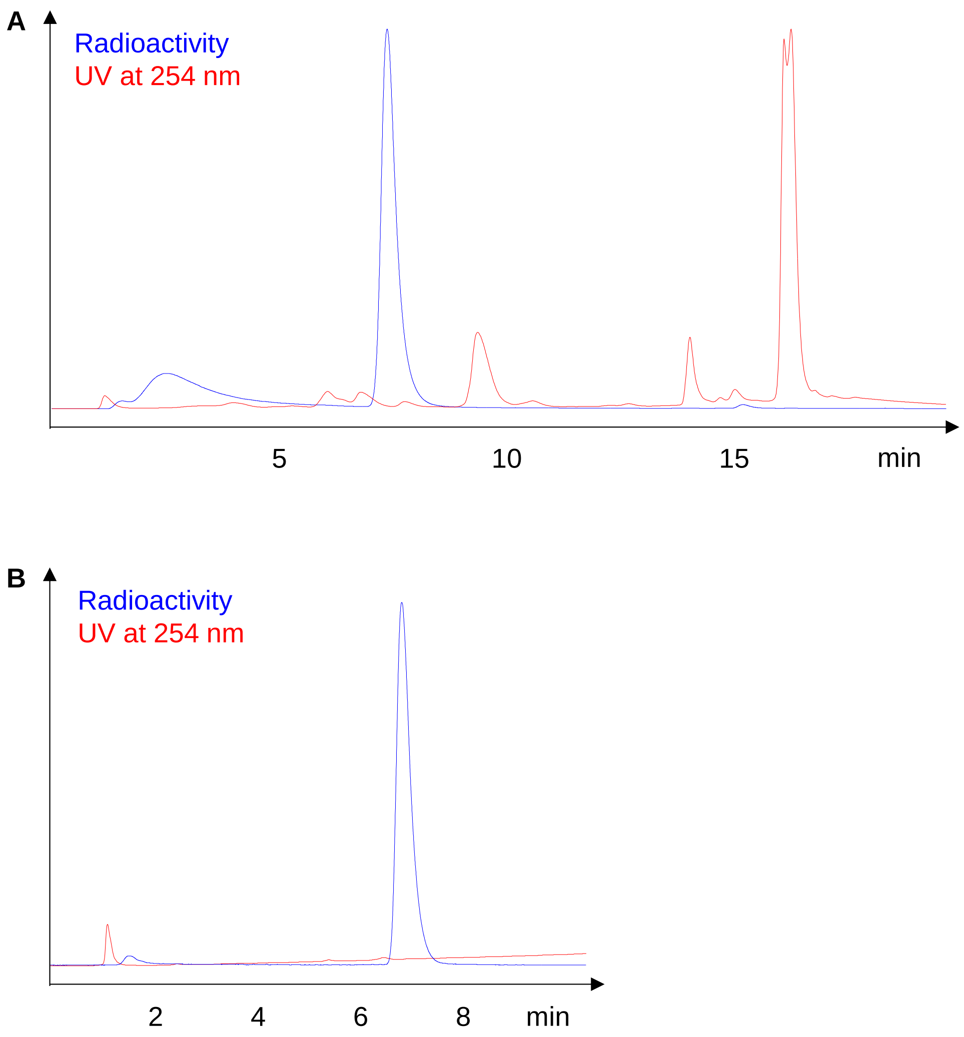

Figure S2. Radio-HPLC trace of [^{18}F]AldoView

Crude reaction mixture (**A**) and isolated product (**B**) on analytical Agilent Eclipse® Plus C18 column

Table S1. [^{18}F]AldoView Binding in Adrenal Surgical Specimens [a]

Case	Disease	Specimen	**TB** [kBq/cm^2]	**NSB** [kBq/cm^2]	**SB** [kBq/cm^2]
1	PHA	1a	20.1	0.4	19.7
		1b (high)	17.9	9.3	8.6
		1b (low)	5.6	0.9	4.8
2	PHA	2a	2.8	0.3	2.5
		2b (high)	20.2	1.3	18.9
		2b (low)	2.4	0.3	2.1
		2c (high)	20.0	1.0	19.1
		2c (low)	3.3	0.3	3.0
3	PHA	3a	7.6	0.7	6.8
		3b (high)	15.5	5.1	10.4
		3b (low)	4.3	1.2	3.0
4	PHA	4a	4.2	0.6	3.6
		4b	18.4	1.9	16.5
5	PHA	5a	5.9	0.8	5.1
		5b	5.1	0.9	4.2
		5c	4.5	1.0	3.5
6	Pheochromocytoma	6a	<LOQ	<LOQ	<LOQ
		6b	5.3	1.3	4.0
7	Adrenal tumor	7a	1.7	1.2	0.5
8	Cushing’s	8a	3.7	0.5	3.2

[a]*Abbreviations*: PHA, primary hyperaldosteronism; TB, total binding; NSB, non-specific binding; SB, specific binding; LOQ, limit of quantification.

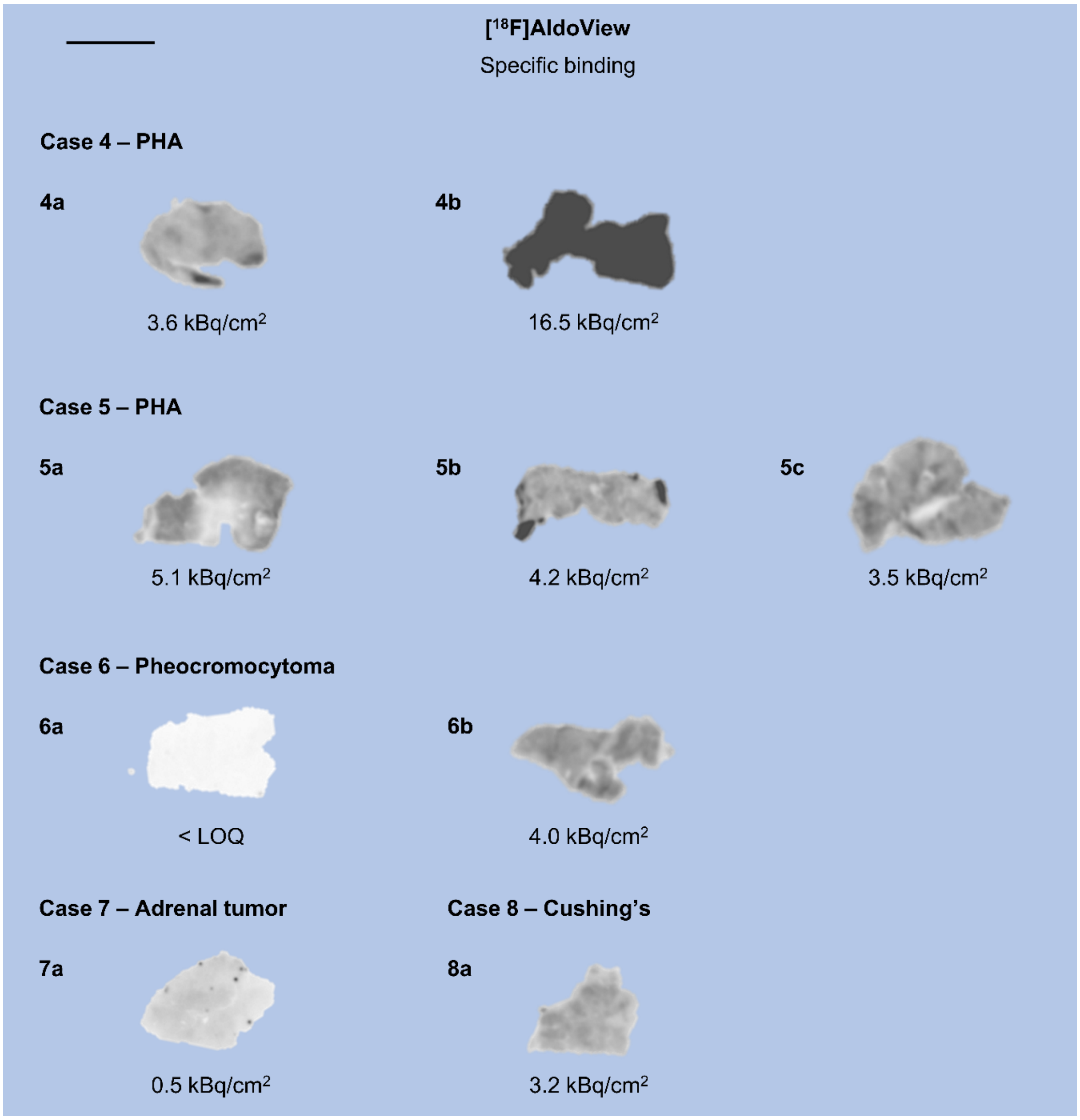

Figure S3. Quantitative phosphorimaging with [^{18}F]AldoView in tissue sections from surgically resected adrenal glands

The images of the tissue sections shown reflect the total tracer binding. Quantification was carried out to obtain the specific [^{18}F]AldoView binding in each surgical specimen. **Case 4** is a male PHA patient aged 67 at time of surgery. Two adenomas (12 mm and 15 mm) were found on CT, one of which (specimen 4a) showed low [^{18}F]AldoView binding, whereas the other one (**4b**) had high [^{18}F]AldoView positivity. In **case 5**, a 67-old male patient, [^{18}F]AldoView binding in the designated APAs (specimens **5a**, **5b**) was 1.2–1.5 times higher than in the adjacent adrenal cortex (specimen **5c**). **Case 6** is a male patient aged 28 at time of surgery, diagnosed with pheochromocytoma. [^{18}F]AldoView binding in the adrenal lesion (specimen **6a**) was below the limit of quantification (LOQ), whereas tracer uptake in the adjacent adrenal cortex (**6b**) was low and comparable to CYP11B2-negative areas of interest. **Case 7** is a 17-year old patient diagnosed with an adrenal tumor with high levels of CYP11B1. Very low [^{18}F]AldoView binding was observed in the tumor section (specimen **7a**). **Case 8** is a patient with Cushing's syndrome, a disease characterized by high CYP11B1, but not CYP11B2 expression. Consistent with this, autoradiography showed low [^{18}F]AldoView binding in the adrenal cortex (specimen **8a**). Scale bar = 1 cm.
